# Treatment for comorbid depressive disorder or subthreshold depression in diabetes mellitus: Systematic review and meta‐analysis

**DOI:** 10.1002/brb3.1981

**Published:** 2020-12-04

**Authors:** Christina van der Feltz‐Cornelis, Sarah F. Allen, Richard I. G. Holt, Richard Roberts, Arie Nouwen, Norman Sartorius

**Affiliations:** ^1^ Department of Health Sciences Hull York Medical School University of York York UK; ^2^ Human Development and Health Faculty of Medicine University of Southampton Southampton UK; ^3^ Department of Family Medicine & Community Health University of Wisconsin Madison WI USA; ^4^ Department of Psychology Middlesex University London UK; ^5^ Association for the Improvement of Mental Health Programmes Geneva Switzerland

**Keywords:** depression, diabetes mellitus, glycemic control, meta‐analysis, systematic review, treatments

## Abstract

**Objective:**

To provide an estimate of the effect of interventions on comorbid depressive disorder (MDD) or subthreshold depression in type 1 and type 2 diabetes.

**Methods:**

Systematic review and meta‐analysis. We searched PubMed, PsycINFO, Embase, and the Cochrane Library for randomized controlled trials evaluating the outcome of depression treatments in diabetes and comorbid MDD or subthreshold symptoms published before August 2019 compared to care as usual (CAU), placebo, waiting list (WL), or active comparator treatment as in a comparative effectiveness trial (CET). Primary outcomes were depressive symptom severity and glycemic control. Cohen's d is reported.

**Results:**

Forty‐three randomized controlled trials (RCTs) were selected, and 32 RCTs comprising 3,543 patients were included in the meta‐analysis. Our meta‐analysis showed that, compared to CAU, placebo or WL, all interventions showed a significant effect on combined outcome 0,485 (95% CI 0.360; 0.609). All interventions showed a significant effect on depression. Pharmacological treatment, group therapy, psychotherapy, and collaborative care had a significant effect on glycemic control. High baseline depression score was associated with a greater reduction in HbA_1_c and depressive outcome. High baseline HbA_1_c was associated with a greater reduction in HbA_1_c.

**Conclusion:**

All treatments are effective for comorbid depression in type 1 diabetes and type 2 diabetes. Over the last decade, new interventions with large effect sizes have been introduced, such as group‐based therapy, online treatment, and exercise. Although all interventions were effective for depression, not all treatments were effective for glycemic control. Effective interventions in comorbid depressive disorder may not be as effective in comorbid subthreshold depression. Baseline depression and HbA_1_c scores modify the treatment effect. Based on the findings, we provide guidance for treatment depending on patient profile and desired outcome, and discuss possible avenues for further research.

## Graphical abstract

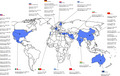

## 1 SUMMATIONS

This systematic review and meta‐analysis exploring psychotherapeutic, pharmacologic, and other interventions shows beneficial treatment effects for comorbid depression in type 1 and type 2 diabetes mellitus with moderate‐to‐large effect sizes for most intervention types.

Although all interventions were effective for depression, not all treatments were effective for glycemic control.

Effective interventions in comorbid depressive disorder may not be as effective in comorbid subthreshold depression.

## 2 LIMITATIONS

Most of the selected studies did not meet all criteria to reduce the risk of bias and not all provided sufficient data to be included in the meta‐analysis.

Further, some treatments were only evaluated in a single RCT.

There is a scarcity of data from many low‐ and middle‐income countries.

## 3 INTRODUCTION

No international consensus exists to guide treatment of comorbid depression in diabetes. Nonetheless, over the last three decades, clinicians have been seeing increasing numbers of patients with comorbid depression of various severity in diabetes (Khaledi et al., 2019; Zheng et al., 2018) due to the exploding prevalence of both diabetes and depression (GBD Disease & Injury Incidence & Prevalence Collaborators, 2018). This can amount to up to 30% depending on severity of symptoms and it occurs especially where the person with diabetes has elevated HbA_1_c despite treatment, or frequent episodes of hypoglycemia and increased glucose variability, diabetes‐related complications, and disengagement from treatments (Groot et al., 2001; Lustman, Anderson, et al., 2000; O'Connor et al., 2009). Depression is a serious psychiatric disorder characterized by loss of interest or pleasure, depressed mood, and suicidal behavior (Ruengorn et al., 2012). Diabetes and depression can both seriously affect an individual's quality of life, and lead to functional disability, increased distress, and social burden (Renn et al., 2011). Depressive symptoms in people with diabetes can have a detrimental impact on engagement with diabetes management (Ciechanowski et al., 2000; Gonzalez, Peyrot, et al., 2008) and on glycemic control (Lustman, Anderson, et al., 2000) as well as on health‐related outcomes (e.g., weight gain and diabetes‐related complications) and associated healthcare costs (Black et al., 2003) As such, the high prevalence of this comorbidity is accompanied by high rates of morbidity and mortality worldwide (Hofmann et al., 2013; Lloyd et al., 2018; Nouwen et al., 2019). Epidemiological studies indicate there is a bidirectional relationship between diabetes and depression (Golden et al., 2008; Katon, 2008; Katon et al., 2007), in which individuals with diabetes have an increased risk of depression and vice versa; the presence of a depressive disorder can increase the risk of metabolic diseases such as diabetes (Renn et al., 2011) and there is an association between depression and diabetes complications (Groot et al., 2001; Van Steenbergen‐Weijenburg et al., 2011).

Evidence is growing to suggest that depression may play a role in the pathogenesis of diabetes in a number of ways. Depression may be a consequence of similar environmental factors that govern glucose metabolism, and can also independently influence nutrition and lifestyle choices which can predispose individuals to the development of diabetes (Beydoun & Wang, 2010). Biological mechanisms have also been proposed through a dysregulated and overactive HPA axis, a shift in sympathetic nervous system tone toward enhanced sympathetic activity, and a pro‐inflammatory state (Champaneri et al., 2010; Joseph & Golden, 2017). The role of inflammation is particularly pertinent. Laake et al. (2014) found that increased inflammation may be involved in the pathogenesis of depression in people with type 2 diabetes, which in turn could contribute to the increased risk of complications and mortality in this clinical population (Geraets et al., 2020).

The relationship between depressive symptoms and poorer diabetes self‐care (Gonzalez, Safren, et al., 2008) applies also to subclinical or subthreshold depressive symptoms (Pibernik‐Okanović et al., 2011) and not only to major depressive disorder. Subthreshold refers to those with two or more depressive symptoms who do not meet the diagnostic criteria for depression (Rodríguez et al., 2012). Subthreshold depressive symptoms in people with diabetes have been found to be persistent but also associated with an increased risk of worsening over time (Bot et al., 2010; Nefs et al., 2012; Pibernik‐Okanovic et al., 2008). Furthermore, an increased incidence of adverse health outcomes and suboptimal metabolic control has been observed not only in patients with the established diagnosis of depression but also in those suffering subthreshold depressive symptoms (Johnson et al., 2014). This indicates that even mild depression is clinically relevant, and implies that combined treatments could also be efficacious for people with diabetes and subthreshold depressive symptoms.

A lack of a clear understanding of the shared origins of depression and diabetes means that finding the most appropriate treatment for this comorbidity in this vulnerable patient group is difficult. In order to optimize health outcomes, feasible and effective interventions aiming to provide benefits to both physical and mental health are recommended (Baumeister & Bengel, 2012; Baumeister et al., 2014; Harkness et al., 2010). The focus of treatment strategies should be on the remission or improvement of depression, in addition to improvement in glycemic control as a marker of diabetes outcome (Petrak et al., 2015).

Evidence shows that comorbid depression in diabetes can be treated with moderate success by psychological and pharmacological interventions, often implemented by using collaborative care (Katon, Von Korf, et al., 2004) and stepped care approaches (Stoop et al., 2015). However, there is conflicting evidence for the efficacy of antidepressants and psychological therapy in the improvement of glycemic control (Lustman, Anderson, et al., 2000; Lustman et al., 1997, 1998a, 2000b, 2007). Petrak, Herpertz, et al. (2015)) claim that more research is needed to evaluate treatment of different subtypes of depression in people with diabetes as well as the effectiveness of new approaches to treatment.

### 3.1 Rationale and objective

A previous systematic review of treatments for comorbid depression in diabetes indicated favorable effects on depressive outcome according to rating scales (Van der Feltz‐Cornelis et al., 2010), but did not include data for subthreshold depression, which has been found to be related to poorer diabetes outcomes similar to DSM‐5 depressive disorder (Gonzalez, Safren, et al., 2008; Pibernik‐Okanović et al., 2011). We updated and expanded this systematic review and meta‐analysis of randomized controlled trials to provide an estimate of the effect of interventions for comorbid depressive disorder or subthreshold depression in type 1 diabetes and type 2 diabetes. The interventions were compared with care as usual (CAU), waiting list (WL), placebo or another active comparator (e.g., another antidepressant or psychotherapy) on depression outcome and glycemic control, and, if possible, to provide treatment guidance for this condition.

## 4 METHOD

This systematic review and meta‐analysis was performed in accordance with the Preferred Reporting Items for Systematic Reviews and Meta‐Analyses (PRISMA) statement (Liberati et al., 2009). We searched MEDLINE, Embase, the Cochrane Central Register of Controlled Trials, and Web of Science using Ovid software. The full search strategy and keywords used have been published elsewhere (Van der Feltz‐Cornelis et al., 2010) and are shown in the appendix (pp 1–2). The reference lists of selected RCTs and reviews were checked for relevant studies that were not included in the databases. The search was supported by the Centre for Reviews and Dissemination at the University of York. The protocol for this review is registered on PROSPERO and can be found here: https://www.crd.york.ac.uk/prospero/display_record.php?ID=CRD42019147910

The final search results were restricted to studies completed before 28th August 2019. Inclusion criteria for studies were that they should be randomized clinical trials, provide a treatment intended to have an effect on both comorbid depressive symptoms and glycemic control in type 1 diabetes and/or type 2 diabetes, and have a control arm (e.g., CAU, placebo, WL or active comparator). The intervention had to be described sufficiently in order to be classified as a psychotherapeutic, medical, pharmacological, collaborative care or other type of intervention. A glossary providing an explanation about the interventions and a list of acronyms are provided in the appendix (pp 3–4).

Participants were adult patients with diabetes and comorbid depressive or subthreshold depression, which was defined as the presence of two or more core depressive symptoms, but not meeting the DSM‐5 diagnostic criteria for depressive disorder (Rodríguez et al., 2012). No restriction was placed on type of intervention or publication language. Studies were not included if depressive disorder or depressive symptoms were not established in a systematic manner such as by semistructured interview or questionnaire at baseline. Studies were selected in a two‐stage process. First, titles and abstracts from the electronic searches were scrutinized by two independent reviewers (SA and CFC). Second, if the abstract met inclusion criteria, we obtained full texts and final decisions were made about study inclusion. Disagreement regarding inclusion status was discussed. Consensus was reached in all cases.

Two reviewers (SA and CFC) independently extracted data for participants’ characteristics, interventions, and study outcomes. A proforma as used in the original systematic review (Van der Feltz‐Cornelis et al., 2010) was used to extract data from the included studies, now also including subthreshold depression from the search hits. The extracted data included: author and year; country; study type; sample size; age; baseline depression measure/diagnostic tool; baseline depression score, baseline glycemic control score, intervention details; control group, length of follow‐up; diabetes and depression outcomes with regard to: i) the change in depression score from baseline to last follow‐up using any validated self‐report measure of depressive symptomatology and ii) the change in levels of biological marker of glycemic control from baseline to last follow‐up. Assessment of glycemic control could be using HbA_1_c, which provides an integrated measure of mean blood glucose levels over the last 6–8 weeks, or FBG, which gives an indication of the blood glucose concentration at the moment of assessment. If both were reported, we used the HbA_1_c to calculate a standardized mean difference. The difference in means of each outcome was the primary measure within each study. Additional outcomes on adherence to recommendations of healthcare providers with regard to self‐care behaviors were extracted if reported. Authors were approached for additional data when questions arose.

### 4.1 Risk‐of‐bias assessment

The Cochrane risk‐of‐bias tool (McGuire et al., 1998) was used to assess random sequence generation (selection bias), allocation concealment (selection bias), blinding of participants and personnel (performance bias), blinding of outcome assessment (detection bias), incomplete outcome data (attrition bias), selective reporting (reporting bias), and other biases. Risk of bias was assessed by SA and CFC independently.

Initial disagreements were resolved by consensus (Appendix pp 6–9). As psychotherapy trials often have limitations in the possibility for blinding (Van der Feltz‐Cornelis & Ader, 2000), studies with limited blinding procedures were not excluded from the analysis. GRADE assessments were made (Guyatt et al., 2008) to give the confidence in each reported effect size. They are reported in the appendix (Appendix page 6–9).

### 4.2 Statistical analysis

As a first step, overall meta‐analysis was performed for all RCTs comparing all treatments with CAU, WL, or placebo for the combined effect on depressive outcome and glycemic control (illness burden). Then, we performed an analysis of illness burden in the studies reporting on depression versus the studies reporting on subthreshold depression. Then, studies were grouped according to the mode of treatment (pharmacotherapy, psychotherapy, collaborative care, online, phone and group interventions, exercise), depression severity (both as depression scores at baseline, and as classification of major depressive disorder or subthreshold depression), and depressive or diabetes outcome. Effect sizes were calculated. Outcomes from individual studies were pooled using a random‐effects model (DerSimonian & Laird, 1986), as this approach assumes that there could be clinical and methodological heterogeneity that might affect the findings. All pooled analyses were reported with 95% confidence intervals (CIs). The effects were presented in terms of standardized effect sizes (Cohen's d). An effect size of 0.5 indicates that the mean of the experimental group is half a standard unit larger than the mean of the control group. It is generally assumed that an effect size of 0.56–1.2 represents a large clinical effect, while effect sizes of 0.33–0.55 are moderate, and effect sizes of 0–0.32 are small (Lipsey & Wilson, 1993). A meta‐regression was conducted to assess whether baseline levels of depressive severity (scores on depression questionnaires) (Appendix pp.15) or glycemic control (HbA_1_c) influenced the effect of the intervention. Between‐study heterogeneity was assessed using the I (Khaledi et al., 2019) statistic (Higgins, 2003). Publication bias was examined by constructing a Begg funnel plot (Begg, 1994) and Duvalls trim and fill (Rothstein et al., 2005). We adhered to published guidance of the Cochrane handbook (Higgins et al., 2019) throughout. We used the statistical program Comprehensive Meta‐Analysis, version 2 (Biostat, 2005) to conduct random‐effects meta‐analyses.

## 5 RESULTS

**Figure 1:**
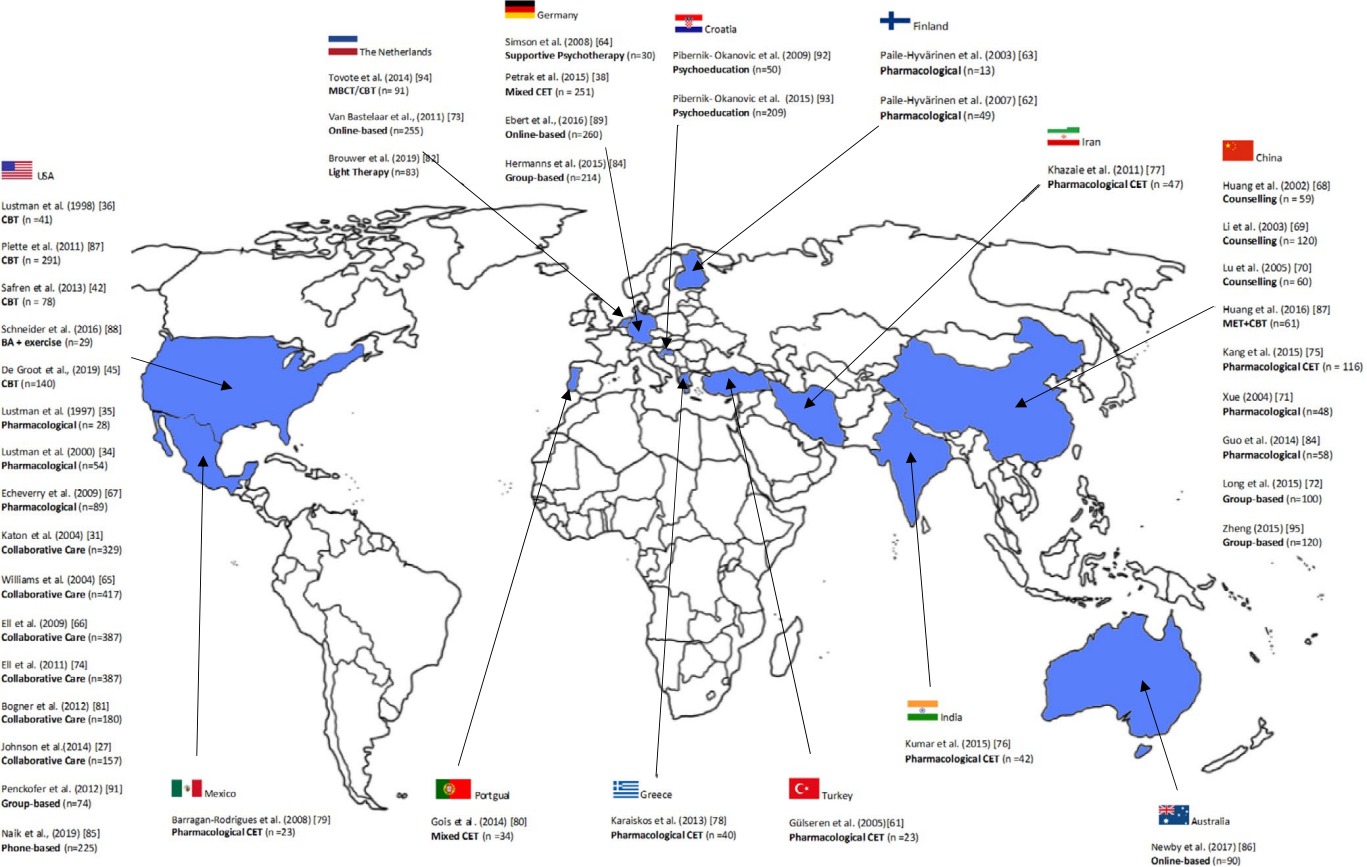
Map of the world showing the location of each study included in the review

A PRISMA flowchart of study selection is presented in the appendix (pp 5). The overall search strategy yielded 8,684 citations of which 43 studies with 4,602 patients were included. This included fifteen studies from the original systematic review (Echeverry et al., 2009; Ell et al., 2010; Gulseren et al., 2005; Huang et al., 2002; Katon, Von Korff, et al., 2004; Li et al., 2003; Lu Xs & Bx, 2005; Lustman, Freedland, et al., 2000; Lustman et al., 1997, 1998b; Paile‐Hyvärinen et al., 2003; Paile‐Hyvarinen et al., 2007; Simson et al., 2008; Williams et al., 2004; Xue, 2004). Of the selected 43 studies, 39 were written or available in English, and four in Chinese (Huang et al., 2002; Li et al., 2003; Lu Xs & Bx, 2005; Xue, 2004). The latter were translated by certified translators and were included in the review. Eight trials with active comparator (CETs) were not entered in the meta‐analysis in accordance with the Cochrane Handbook instructions for dealing with heterogeneity (Higgins et al., 2019), as pooling was not possible because of heterogeneous control groups (Barragán‐Rodríguez et al., 2008; Gois et al., 2014; Gulseren et al., 2005; Kang et al., 2015; Karaiskos et al., 2013; Khazaie et al., 2011; Kumar et al., 2015; Petrak, Herpertz, et al., 2015). Three RCTs were not entered because they did not present the data required for pooling (Bastelaar et al., 2011; Brouwer et al., 2019; Ell et al., 2011). Thirty‐two RCTs with 3,543 patients with type 1 diabetes and type 2 diabetes were entered into the meta‐analysis (Bogner et al., 2012; Ebert et al., 2017; Echeverry et al., 2009; Ell et al., 2010; Groot et al., 2019; Guo et al., 2014; Hermanns et al., 2015; Huang et al., 2002, 2016; Johnson et al., 2014; Katon, Von Korff, et al., 2004; Li et al., 2003; Long et al., 2015; Lu Xs & Bx, 2005; Lustman, Freedland, et al., 2000; Lustman et al., 1997, 1998b; Naik et al., 2019; Newby et al., 2017; Paile‐Hyvärinen et al., 2003; Paile‐Hyvarinen et al., 2007; Penckofer et al., 2012; Pibernik‐Okanovic et al., 2009, 2015; Piette et al., 2011; Safren et al., 2014; Schneider et al., 2016; Simson et al., 2008; Tovote et al., 2014; Williams et al., 2004; Xue, 2004; Zheng et al., 2015) All studies were performed in patients with diabetes as the primary or index condition, who suffered from comorbid depressive disorder or subthreshold depression. The countries in which each study was conducted are shown in Figure 1.

Results are shown in Table 1. The studies reported mostly on type 2 diabetes, or on type 1 and type 2 diabetes combined.

**Table 1:** Data extraction table (*N* = 43)

Author (year)	*n* (completers) Mean age	Measure for depression classification	Intervention conditions and follow‐up	Baseline depression, diabetes (Mean, *SD*)	Outcome assessment; depression, diabetes	Effect size depression, diabetes	Comments	MDD/sub thres‐hold	Add‐on exercise		Adherence focus
Psychotherapeutic interventions (11 RCTs, *n* = 1,010)											
Lustman et al. (1998) USA	*n* = 41 Type 2 DM 100% 53.1–56.4 ± 10.5–9.7	MDD according to DIS and BDI ≥ 14	CBT plus diabetes education versus diabetes education alone (CAU) FU: 11 wk, 6 months	*Depression:* BDI: CBT = 24.9 (10.2); Control = 21.1(6.8). *Diabetes:* HbA_1_c: CBT = 10.2(3.6); Control = 10.4 (3.1)	*Depression*: Response (reduction BDI ≥ 50%) *p*<.001 in CBT group *Diabetes:* HbA_1_c lower in CBT group *p*<.03	*Depression:* Δ − 1.112 *Diabetes:* Δ − 0.704	Improvement in depression as well as glycemic control in CBT versus. control	MDD	No		No
Huang et al. (2002) China	*n* = 59 Type 2 DM 100%	SDS > 50	Antidiabetics + diabetic education + psychological treatment + relaxation and music treatment versus. Antidiabetics only (CAU) FU: 3 months	*n*/a	*Depression*: SDS total score difference in means 0.07; *p*<.05 *Diabetes:* HbA_1_c difference in means 1.7; *p*<.05	*Depression:* Δ − 0.521 *Diabetes:* Δ − 0.521	Improvement in depression as well as glycemic control in CBT versus. control.	MDD	No		No
Li et al. (2003) China	*n* = 120 Type DM % not stated 50.5–52.3 ± 10.4–11.2	SDS ≥ 50	Antidiabetics + diabetic education + psychological treatment versus. antidiabetics only (CAU) FU: 4 wk	*n*/a	*Depression*: SDS total score difference in means 13.4, *p*<.01 *Diabetes*: FBG difference means 2.09, *p*<.05	*Depression* Δ − 0.478: *Diabetes*: Δ − 0.362	Anxiety (SAS ≥ 50) taken into account as well. Improvement in depression as well as glycemic control in CBT versus. control	MDD	No		No
Lu et al. (2005) China	*n* = 60 Type 2 DM 100% 65.6–64.9 ±9.8–9.5	Mental maladjustment caused by CVA according to the CCMD−2‐R and HAMD−17 ≥ 8	Diabetes and CVA education + electromyographic treatment + psychological treatment versus. CAU FU: 4 wk	*Depression*: HAMD: study group = 16.2 (5.7) control group = 16.8 (5.1). FBG: study group = 9.76 (3.83); control group = 9.89(3.94). *Diabetes:* 2HPG: study group = 13.65(4.72); control group = 13.31(4.57).	*Depression*: HAMD−17 total score difference in means 7.3; *p*<.01 *Diabetes*: difference in means FPG 1.54; *p*<.05	*Depression* Δ − 0.688: *Diabetes*: Δ − 0.517	Hemiplegia after CVA as DM complication. Improvement in depression as well as glycemic control in CBT versus. control	MDD	No		No
Simson et al. (2008) Germany	*n* = 30 Type 1 and Type 2 DM 80% 60.5 (±10.9)	HADS depression score ≥ 8	Individual supportive psychotherapy versus. CAU FU: discharge (3– 20 wk)	*Depression:* HADS‐D: Psychotherapy = 11.7 (2.7); Control = 10.6 (2.9). *Diabetes*: HbA_1_c: Psychotherapy = 7.8 (1.5); Control = 8.7 (1.8).	*Depression*: HADS depression scale total score mean difference 1.9; *p*=.018 *Diabetes*: PAID mean difference 7.6; *p*=.008	*Depression*: Δ − 0.918 *Diabetes*: Δ−1.043	Diabetic foot as DM complication; Inpatients. Improvement in depression as well as glycemic control in supportive psychotherapy versus. control	MDD	No		No
Piette et al. (2011) USA	*n = *291 Type 2 DM = 100% 56.0 (±10.1)	BDI ≥ 14	Telephone delivered CBT plus walking program versus Enhanced CAU FU: 12 months.	*Depression*: BDI: EUC = 26.5 (9.9); CBT = 26.7 (7.7) *Diabetes*: HbA_1_c: EUC = 7.7(1.7); CBT = 7.5 (1.7).	*Depression*: BDI total score mean between group difference −4.5, *p*<.0001 *Diabetes*: HbA_1_C mean between group difference 0.07, *p*=.70..	*Depression:* Δ0.418 *Diabetes*: Δ0.000	Improvement in depressive symptoms but not glycemic control for telephone CBT + walking versus. control	MDD	Yes		No
Safren et al. (2014) [89] USA	*n = *78 Type 2 DM = 100% 55.44–58.31 (±8.72–7.41)	MDD as defined by DSM‐IV assessed by clinician using MINI	CBT for adherence and depression versus enhanced CAU FU: 4, 8 and 12 months	*Depression:* MADRS: CBT‐AD = 25.6(8.99); ETAU = 23.31(7.20). *Diabetes*: HbA_1_c: CBT‐AD = 8.81(1.78); ETAU = 8.74(1.41).	*Depression*: MADRS score mean difference 6.22 (*p*=.002). CGI ratings mean difference 0.74, (*p*=.01) *Diabetes*: HbA_1_C mean difference 0.72, *p*=.001.	*Depression:* Δ0.762 *Diabetes*: Δ 2.311	Main focus on adherence. Significant improvements in depression as well as glycemic control in CBT‐AD versus. control.	MDD	No		Yes
Tovote et al. (2014) Netherlands	*n* = 91 Type 2 DM = 61% Mean age = 53.1 (±11.8)	BDI‐II ≥ 14	8‐weekly sessions of Mindfulness based cognitive therapy versus CBT versus wait list control. FU: 3 months	*Depression:* BDI‐II: MBCT = 23.6(7.7); CBT = 25.6(8.7); control = 24.3(8.0); HAM‐D7: MBCT = 8.9(3.5); CBT = 9.4 (3.8); control = 7.5(2.8). *Diabetes:* HbA_1_c: MBCT = 8.0(0.9); CBT = 8.3(1.4)	*Depression:* BDI‐II scores and HAM‐D7 showed significant improvements in both interventions (*p*<.001). There was a clinically relevant improvement of 26% (MBCT) and 29% (CBT) versus. 4% (control). *Diabetes:* HbA_1_c levels did not change significantly after MBCT (*p*=.92) or CBT (*p*=.72)	MBCT: *Depression:* Δ 0.568 *Diabetes:* *n*/a CBT: *Depression:* Δ 0.541 *Diabetes:* *n*/a	Significant improvement in depressive symptoms for both MBCT and CBT versus wait list control. HbA1c levels did not improve in either intervention group.	Sub	No		No
Schneider et al. (2016) USA	*n* = 29 Type 2 DM = 100% 53.4 (±7.1) 100% female	MDD as defined by SCID‐IV	Behavioral action with exercise versus enhanced CAU. FU: 3 and 6 months	*Depression*: BDI‐II: EUC = 21.6 (4.7); EX = 18.5 (8.2); HDSR: EUC = 17.4 (4.3); EX = 15.7(4.6) *Diabetes*: HbA_1_c: EUC = 7.9(0.6); EX = 7.9 (0.8).	*Depression*: BDI‐II total score mean difference −7.3, *p*<.0001, HRSD mean difference score = −6.6, (*p*<.0001). *Diabetes*: Time x condition was not significant for HbA_1_c (*p* = .78).	*Depression:* Δ−0.018 *Diabetes*: Δ−0.114	Significant improvement in depressive symptoms in both EX group and EUC group. No improvement in glycemic control.	MDD	Yes		No
Huang et al. (2016) China	*n = *61 Type 2 DM = 100% 56.43 (±10.42)	CES‐D=>16 (indicating “significant” or mild depressive symptomatology)	Combined behavioral intervention 12 sessions over 3 months versus CAU FU: 3 months	*Depression*: CES‐D: CBT + MET =21.81 (5.68); EUC = 21.97 (3.37). *Diabetes:* HbA_1_c: CBT + MET =7.68(1.44); EUC = 7.84 (1.95).	*Depression*: CES‐D scores reduced significantly (−6.28, *p*<.01) in intervention group. *Diabetes:* HbA_1_c levels reduced significantly in the intervention group (−4.86) and were significantly lower in the intervention group than control (*p*<.01).	*Depression:* Δ 2.298 *Diabetes*: Δ0.915	Improvement in depressive symptoms and glycemic control for MET + CBT versus. control	Sub	No		No
De Groot et al. (2019) USA	*n* = 140 Type 2 DM = 100% 56.0 (±10.7)	Structured clinical interview for DSM_IV‐TR, BDI‐II	CBT alone versus community‐based exercise versus CBT + exercise versus CAU FU: 12 weeks	*Depression:* BDI‐II: (values not reported). *Diabetes:* HbA_1_c: CBT = 8.0(1.6); EX = 8.1(1.7); CBT + EX=7.5(1.6); UC = 8.0(1.9).	*Depression:* Full remission from MDD was 66% in CBT, 72% in Ex, 71% in CBT + Ex compared with 32% in CAU. BDI‐II scores lower in all three groups compared to CAU (ps<0.05). *Diabetes:* HbA_1_c levels lower for CBT + ex (*p*=.0016), but not CBT (*p* = .379) or Ex alone (*p* = .132)	CBT: *Depression:* Δ 0.678 *Diabetes:* Δ0.274; Exercise: *Depression:* Δ 0.640; *Diabetes:* Δ 0.467; CBT + Ex: *Depression:* Δ 0.671; *Diabetes:* Δ0.912.	Significantly larger improvement in depressive symptoms in CBT alone, exercise alone and CBT + exercise compared with CAU. Glycemic control only showed significant improvements in CBT + exercise group versus CAU.	MDD	Yes		No
Comparison of different pharmacological agents (6 RCTs, comparative effectiveness trials (CETs) *n* = 291)											
Gülseren et al. (2005) Turkey	*n* = 23 Type 2 DM 100% 58.2–57.1 ± 12.3–10.4	MDD according to SCID, HADS depression subscale score ≥ 10, HDRS ≥ 16	Fluoxetine versus. paroxetine FU: 12 wk	*Depression:* HDRS: Fluoxetine = 17.5 (2.4); Paroxetine = 18.8(3.0). *Diabetes:* HbA_1_c: Fluoxetine = 6.9(1.7); Paroxetine = 6.9(1.2)	*Depression*: Both groups improved significantly in HDRS scores (mean difference 0.62; *p*=.003) Diabetes: No difference in HbA1c (mean diff 0.11; n.s.)		No significant difference between both conditions. This study is not pooled in the meta‐analysis.	MDD	No		No
Barragan‐Rodrigues et al. (2008) Mexico	*n* = 23 Type 2 DM = 100%, 66.4–69 (±5.9–6.1)	Ysavage and Brink score > 11.	Magnesium supplementation versus imipramine *FU:* 12 weeks	*Depression*: Ysavage & Brink: MgCl2 = 17.9(3.9); Imipramine = 16.1(4.5). *Diabetes*: HbA_1_c: MgCl2 = 8.9(1.6) Imipramine = 9.0(1.7); FBG: MgCl2 = 194.3 (59.0); Imipramine = 183.4(68.0).	*Depression:* Ysavage and brink scores reduced for MgCL2 (−6.5; *p*<.005) and imipramine (−6.2; *p*<.005). *Diabetes:* No change for HbA1c or FBG levels.	*n*/a	Depressive symptoms but not glycemic control improved in both MgCl2 and imipramine groups. No control group so study not included in meta‐analysis.	MDD	No		No
Khazaie et al. (2011) Iran	*n* = 47 Type 2 DM = 100% 47.7–51.57 (±8.63–8.38)	BDI ≥ 14	Fluoxetine versus Citalopram FU:12 weeks	*Depression:* BDI: Fluoxetine = 29.29 (3.50); Citalopram = 25.26(3.51). *Diabetes:* HbA_1_c: Fluoxetine = 7.68 (1.69); Citalopram = 8.25 (1.34); FBG: Fluoxetine = 159.21 (39.66); Citalopram = 163.37 (49.24).	*Depression:* BDI scores improved for fluoxetine (−15.12) *p*<.001) and citalopram (11.84, *p*<.001). *Diabetes:* HbA_1_c levels improved for fluoxetine (−1.94; *p*<.001) and citalopram (−1.59; *p*<.001). FBG also improved in Fluoxetine (48.93; *p*<.001) and citalopram (39.95; *p*<.001).	*n*/a	Fluoxetine and Citalopram both improved depression symptoms and glycemic control. No control group so study not included in meta‐analysis.	Sub	No		No
Karaiskos et al. (2013) Greece	*n* = 40 Type 2 DM = 100% 52.4–54.3 (±11.4–12.5)	Classification of mood disorder based on DSM‐IV‐TR criteria	Agomelatine versus Sertraline *FU*:4 months	*Depression*: HDRS: Agomelatine = 11.6(2.5); Sertraline = 11.5(2.5). *Diabetes:* HbA_1_c: Agomelatine = 7.7(0.5); Sertraline = 7.6(0.5); FBG: Agomelatine = 137(21); Sertraline = 135(16).	*Depression*: HDRS scores reduced more for Agomelatine (−5.8) than sertraline (−4.2) (*p* = .050). *Diabetes*: No effect on FPG levels (*p* = .694). HbA1c levels reduced more for Agomelatine (−0.5) than sertraline (−0.0) (*p* = .044).	*n*/a	Depressive symptoms and HbA1c levels but not FBG levels were significantly lower in Agomelatine group compared to the sertraline group at follow‐up. No control group so study not included in meta‐analysis.	MDD	No		No
Kang et al. (2015) China	*n* = 116 Type 2 DM = 100% 50.82–52.50 (±11.36–10.27)	HDRS ≥ 17 (indicating moderate‐to‐severe depression) + psychiatrist's assessment according to DSM criteria.	Agomelatine versus paroxetine FU: 6,12 weeks	*Depression:* HDRS: Paroxetine = 23.94(3.07) Agomelatine = 24.20(3.38) *Diabetes:* HbA_1_c: Paroxetine = 7.71, Agomelatine = 7.84 (0.45).	*Depression*: HDRS scores improved for Agomelatine (−14.85) and paroxetine (−13.44) (ps<0.001). *Diabetes*: HbA_1_c levels significantly lower for Agomelatine (Δ −0.62, *p*<.001) but not paroxetine (*p*=.038).	*n*/a	Both drugs significantly improved depressive symptoms. Agomelatine better than paroxetine for glycemic control. No control group so study not included in meta‐analysis.	MDD	No		No
Kumar et al. (2015) India	*n* = 42 Type 2 DM = 100% 48.65–49.75 (±10.19–14.27)	HDRS ≥ 14	Agomelatine versus Escitalopram FU: 1 month, 2 months	*Depression:* HDRS: Escitalopram = 18.5 (2.95) Agomelatine = 17.15(2.54) MADRS: Escitalopram = 22.95(2.74) Agomelatine = 17.15(2.54) *Diabetes:* HbA_1_c: Escitalopram = 7.2 (0.36) Agomelatine = 7.35 (0.41) FBG: Escitalopram = 131.9 (3.45) Agomelatine = 132.3 (4.41)	*Depression:* Larger improvement in Escitalopram group for HDRS scores (−9.65 v −1.55, *p*<.001); and MADRS scores (−9.35 v. −2.00, *p*<.001) *Diabetes:* Larger HbA_1_c improvement in Escitalopram (−0.09) than Agomelatine (−0.03) (*p*=.047). Larger reduction in FBG in Escitalopram (−6.95) than Agomelatine (−4.45) (*p*=.043).	*n*/a	Escitalopram appears to be better than Agomelatine for improving both depression and glycemic control. No control group so study not included in meta‐analysis.	Sub	No		No
Pharmacological versus psychotherapeutic (2 RCTs, CETs, *n* = 149)											
Gois et al. (2014) Portugal	*n* = 34 Type 2 DM = 100% 55.14 (±5.92)	HADS > 7; MADRS > 17 & MDD according to MINI	Sertraline and clinical management versus Interpersonal psychotherapy FU: 6 weeks, 14 weeks, 24 weeks	*Depression:* MADRS: Sertraline = 24.64(6.4); IPT = 26.45 (4.37). *Diabetes:* HbA_1_c: Sertraline = 9.28(2.67); IPT = 8.69(2.20).	*Depression:* MADRS scores improved in both IPT (−14.00) and sertraline (−14.00) (ps<0.0001). *Diabetes:* No significant improvement in HbA_1_c levels.	*n*/a	No differences in improvements in depressive symptoms between IPT and sertraline. No significant effect on glycemic control was shown for either intervention.	MDD	No		No
Petrak, Baumeister, et al. (2015) Germany	*n* = 251 (*n* = 115) Type 2 DM = 48.6% 48.5 1(±1.7)	CES‐D > 22	Diabetes specific CBT versus sertraline FU: 12 weeks (phase 1), 15 months (excluding nonremitting patients at phase 1)	*Depression:* HAMD−17: CBT = 18.04(4.62); Sertraline = 18.87(5.14); *Diabetes:* HbA_1_c: CBT = 9.37(1.63); Sertraline = 9.15(1.37).	*Depression:* HAMD−17 scores improved in CBT (−10.21) and sertraline (−13.41). Sertraline improvement was significantly better (*p* >. 05). *Diabetes*: No significant change in HbA_1_c levels.	*n*/a	Sertraline and CBT both improve depression after 12 weeks. Significant advantage of sertraline over diabetes specific CBT for improving depressive symptoms over one year, but not glycemic control.	MDD	No		No
Pharmacological treatment versus placebo (7 RCTs, n = 339)											
Lustman et al. (1997) USA	*n* = 28 Type 1 and Type 2 DM 50% 49.0–49.2 ± 12.1–13.7	MDD according to DIS	Glucometer training + nortriptyline versus. placebo FU: 9 wk	*Depression:* BDI: Nortriptyline = 19.0 (7.4); Placebo = 17.8 (7.1) *Diabetes*: HbA_1_c: Nortriptyline = 11.8 (2.9) Placebo = 11.6 (3.1) (*Depressed group *n* = 28)	*Depression*: BDI total score, mean difference 5.6; *p*=.03 *Diabetes*: HbA_1_c, no significant difference, no outcome reported.	*Depression*: Δ − 0.868 *Diabetes*: Δ 0	Poorly controlled (HbA1c ≥ 9%) as inclusion criterion. Improvement in depression but not in glycemic control in nortriptyline versus. control. Nortriptyline may have negative impact on glycemic control.	MDD	No	No	
Lustman, Anderson, et al. (2000) USA	*n* = 54 Type 1 and Type 2 DM 55.6% 45.0–47.7± 13.0–11.5	MDD (DIS), and BDI or HAMD ≥ 14	Fluoxetine versus. placebo FU: 8 wk	*Depression*: BDI: Fluoxetine = 23.6 (8.2); Placebo = 22.4 (9.1); HAMD: Fluoxetine = 20.1 (5.6); Placebo = 19.5 (6.9). *Diabetes*: HbA_1_c: fluoxetine = 8.4 (1.7); Placebo = 8.6 (1.6).	*Depression*: HAMD total score mean difference 26.7; *p*<.04 *Diabetes*: HbA_1_c mean difference 0.33; *p*=.13 (n.s.)	*Depression*: Δ − 0.573 *Diabetes*: Δ 0.419	Improvement in depression but not in glycemic control in fluoxetine versus. placebo.	MDD	No	No	
Paile‐Hyvärinen et al. (2003) Finland	*n* = 13 Type 2 DM 100% 61.1–62.3 ±8.6–11.5	MADRS score between 2.5 and 12 (mild‐to‐moderate depression)	Paroxetine versus. placebo FU: 4 wk	Depression: MADRS: Placebo = 6.4 (4.0); Paroxetine = 7.4(2.9); BDI: Placebo = 13.0 (9.2); Paroxetine = 13.7 (7.4). Diabetes: HbA_1_c: Placebo = 6.9 (0.4); Paroxetine = 7.5(0.8)	After initial improvement in Paroxetine group at 3 months, no significant improvement for both outcomes at end of follow‐up. *Depression*: MADRS total score mean difference 2.50; *p*=.25 (n.s.) *Diabetes*: GHbA_1_c mean difference 0.37; *p*=.08 (n.s.)	*Depression*: Δ − 0.676 *Diabetes*: Δ 1.073	Poorly controlled (HbA1c ≥ 6.5% or FBG ≥ 7.0) as inclusion criterion. Probably a combination of ceiling effect and underpowered study.	MDD	No	No	
Xue (2004) China	*n* = 48 Type 1 and Type 2 DM 85.4% 21–65 age range		Paroxetine versus. placebo FU: 8 wk	*Depression:* HAMD: Paroxetine = 20.1(12.7; control = 19.5 (12.1); BDI: Paroxetine = 23.6(14.2); control = 22.4(15.9). *Diabetes:* GHb: paroxetine = 8.8(1.8); control = 8.7(1.6).	*Depression*: HAMD−17 total score mean difference 5.7; *p*<.01 *Diabetes*: HbA_1_c mean difference 0.4; *p*=.245 (n.s.)	*Depression*: Δ − 0.776 *Diabetes*: Δ 0.340	Improvement in depression but not in glycemic control in paroxetine versus. placebo.	MDD	No	No	
Paile‐Hyvärinen et al. (2007) Finland	*n* = 49 Type 2 DM 100% 59.5–59.2 ± 6.0–5.4	Diagnostic interview. Mild depression (< 6 depressive symptoms according to the *DSM‐IV*).	Paroxetine versus. placebo FU: 3 months, 6 months	*Depression:* HADS‐D: Placebo = 8.4 (3.4); Paroxetine = 7.3 (3.4). *Diabetes:* Placebo = 8.7(1.3); Paroxetine = 8.5 (0.9).	*Depression*: HADS depression scale total score mean difference 0.7; *p*=.448 (n.s.). *Diabetes*: GHbA_1_c mean difference 0.13; *p*=.693 (n.s.	*Depression*: Δ − 0.260 *Diabetes*: Δ 0.135	No significant improvement in depressive outcomes and glycemic control.	MDD	No	No	
Echeverry et al. (2009) USA	*n* = 89 *n* = 87 Type 2 DM; *n* = 2 Type 1 52–53 ±8–10	MDD according to CDIS	Sertraline versus. placebo	*Depression:* HAM‐D: Sertraline = 19.0(5.0); Control = 20.0 (6.0); *Diabetes:* HbA_1_c: Sertraline = 10.0(1.8); Control = 9.7(1.6).	*Depression*: HADS depression scale total score mean difference 1.0; (n.s.). *Diabetes*: GHbA_1_c mean difference 1.1; *p*<.011.0; (n.s.).	*Depression*: Δ − 0.283 *Diabetes*: Δ − 0.480	Significant improvement in depression in both sertraline and placebo; no difference between conditions. Significant improvement in glycemic control in sertraline compared to placebo.	MDD	No	No	
Guo et al. (2014) China	*n* = 58 Type 2 DM = 100% 53.3–54.7(±7.3–7.3)	Classification of depression based on DSM‐IV criteria	Metformin versus placebo FU: 24 weeks	*Depression*: MADRS: Metformin = 23.7 (3.5), placebo = 24.3 (3.8); HRSD17: Metformin = 20.1 (3.0); placebo = 20.4 (2.4). *Diabetes:* HbA_1_c: Metformin = 7.82(0.82); placebo = 8.01(0.59).	*Depression*: MADRS (*p*<.001) and HRSD−17 (*p*<.001) scores both improved for Metformin *Diabetes:* HbA_1_c levels improved compared to placebo group (−1.52 versus 0.19 *p*<.001).	*Depression:* Δ 0.900 *Diabetes:* Δ 3.676	Significant improvement in depressive symptoms and glycemic control in metformin group compared to placebo group.	MDD	No	No	
Psychoeducation (2 RCTs, *n* = 259)											
Pibernik‐ Okanovic et al. (2009) Croatia	*n* = 50 Type 2 DM = 100% Median age = 55 (51–62)‐ 58 (53–64)	Mild‐to‐moderate depression. PHQ9 scores 10–14	Psychoeducation comprising 4 x interactive group meetings versus CAU (Depression screening followed by standard diabetes treatment) *FU:* 6, 12 months	*Depression:* CES‐D: PsyEd = 26(22–30); CAU = 24 (18–35). *Diabetes:* HbA_1_c: PsyEd = 7.5(6.4–8.3); CAU = 7.7(6.6–8.9). *medians (CI)	*Depression:* Median CES‐D scores reduced in both groups. Between group difference n.s (*p*=.074) *Diabetes:* HbA_1_c levels reduced, between group difference n.s (*p*=.089).	*Depression*: Δ 0.135 *Diabetes:* Δ −0.049	Psychoeducation shows no significant benefit for either depressive symptoms or glycemic control over care as usual.	Sub	No		No
Pibernik‐ Okanovic et al. (2015) Croatia	*n* = 209 Type 2 DM = 100% 57.7–58.5 (±6.2–5.6)	PHQ2 ‐ > one depressive symptom over last month	Psychoeducation versus psychoeducation and physical exercise) versus enhanced CAU *FU*: 12 months	*Depression*: CES‐D: PsyEd = 19.7(9.1); PsyEd + Ex =19.8(8.2); CAU = 19.0(8.6). *Diabetes*: HbA_1_c: PsyEd = 7.4(1.3); PsyEd + Ex =7.2(1.0); CAU = 7.1(1.0).	*Depression*: CES‐D improved for all groups (*p*=.003) but not significantly between groups (*p* = .656) *Diabetes:* No significant effects on HbA_1_c levels	PsyEd: *Depression:* Δ0.082 *Diabetes*: Δ−0.210. PsyEd + Ex: *Depression:* Δ−0.074 *Diabetes:* Δ −0.199	Psychoeducation and psychoeducation + exercise showed no significant benefits over treatment as usual for depressive symptoms or glycemic control.	Sub	Yes		No
Collaborative Care (6 RCTs, *n* = 1,133)											
Katon, Von Korf, et al. (2004) USA	*n* = 329 Type 1 and Type 2 DM 95.7% 58.1–58.6 ± 12.0–11.8	PHQ−9 ≥ 10 and SCL−90 depression mean item score > 1.1	Collaborative care versus. CAU FU: 6 months, 12 months		*Depression*: SCL−20 total score mean difference response (reduction SCL−90 ≥ 40% or ≥ 50%) *p*=.004 *Diabetes*: HbA_1_c mean difference 0, n.s.	*Depression*: Δ − 0.320 *Diabetes*: Δ 0.085	Improvement in depression but not in glycemic control in collaborative care versus. usual care.	MDD	No		No
Williams et al. (2004)USA	*n* = 417 Type 1 and Type 2 DM % not stated, mostly Type 2 71.2 ± 7.5	MDD or dysthymia according to SCID	Education about late‐life depression + collaborative care versus. CAU FU: 3 months, 6 months, 12 months		*Depression*: SCL−20 total score mean difference − 0.3; CI − 0.57 to 0.29 *Diabetes*: HbA_1_c mean difference 0, n.s.	*Depression*: Δ − 0.676 *Diabetes*: Δ 0.000	Improvement in depression but not in glycemic control in collaborative care versus. usual care.	MDD	No		No
Ell et al. (2010) USA	*n* = 387 Type 1 and Type 2 DM % group not stated 72% >50 years	PHQ−9 ≥ 10	Collaborative care versus. CAU FU: 6 months, 12 months, 18 months		*Depression*: SCL−20 total score mean difference 50% improved in 62 versus. 44%; *p*<.001 *Diabetes*: HbA_1_c mean difference 0, n.s.	*Depression*: Δ − 0.337 *Diabetes*: *Δ − 0.263*	Significant improvement in depression but not in glycemic control in collaborative care versus. usual care in Hispanics with baseline HbA1c > 8	MDD	No		No
Ell et al. (2011) USA	*n* = 387 Type 2 DM = 98% 54 (±8.7)	PHQ9 scores > 10	Socioculturally adpated collaborative care (MDDP; *n* = 193) versus enhanced CAU (EUC: *n* = 194). FU: 6, 12, 18, 24 months	Depression: SCL−20; PHQ9 (values not reported). Diabetes: HbA_1_c (values not reported).	*Depression:* SCL−20 and PHQ 9 scores improved significantly more in intervention group (ps <0.001). *Diabetes:* No differences in HbA_1_c levels (ps>0.05).	*n*/a	Significantly larger improvements in depressive symptoms were observed in the MDDP group versus care as usual, however these group differences narrowed over time. No effects on glycemic control. Study not entered in meta‐analysis due to lack of data.	MDD	No		No
Bogner et al. (2012) USA	*n* = 180 Type 2 DM = 100% 57.1–57.8 (9.6–9.4)	PHQ9	Integrated care versus CAU FU: 6 and 12 weeks	*Depression*: PHQ9: IC = 10.6(7.9); CAU = 9.9(7.2). *Diabetes*: HbA_1_c: IC = 7.2(1.8); CAU = 7.0(1.9).	*Depression*: PHQ−9 scores improved significantly more in IC group (–2.42; *p*= .007). IC group were more likely to achieve remission (58.7% versus 30.7%; *p*<.001) *Diabetes:* HbA_1_c levels significantly improved in IC group (–0.70 *p* <.001).	Depression: Δ 0.405 *Diabetes*: Δ 0.497	Improvement in glucose control and depressive symptoms in integrated care intervention versus usual care.	MDD	No		Yes
Johnson et al. (2014) [28] USA	*n* = 157 Type 2 DM = 100% 57.0–59.2 (±10.5–8.5)	PHQ scores > 10	TEAMCare (*n* = 95) collaborative care intervention versus screening and follow‐up CAU (control; *n* = 62). *FU*: 6 months, 12 months	*Depression*: PHQ9: TEAMCare = 14.5(3.8): Control = 14.6(3.5). *Diabetes:* HbA_1_c: TEAMcare = 7.5(1.8); Control = 7.8(1.7).	*Depression:* PHQ9 scores improved significantly more in TEAMcare group (−7.3. (*p*=.015). *Diabetes:* No differences in HbA_1_c levels	*Depression:* Δ 0.388 *Diabetes*: Δ 0.244	Significant improvement in depressive symptoms but not glycemic control in collaborative care group versus active control.	MDD	No		No
Online‐based interventions (3 RCTs, *n* = 605)											
Van Bastelaar et al., (2011) Netherlands	*n* = 255 Type 2 DM = 55% 50 (±12)	CES‐D > 16	Web‐based CBT (iCBT; *n* = 125) versus waiting list control (WL; *n* = 130) group. FU:1 month	*Depression*: CES‐D: iCBT = 29(7); WL = 28(7); *Diabetes*: HbA_1_c: iCBT = 7.4(1.6); WL = 7.3(1.4).	*Depression:* Treatment x time interaction effect on CES‐D scores (*p*<.001) was significant. *Diabetes*: No significant treatment effect found for HbA_1_C levels (*p* >.05).	*n*/a	Significant improvement in depressive symptoms but not glycemic control in web‐based‐ CBT group versus active control. Study not entered in meta‐analysis due to lack of reported data.	MDD	No		No
Ebert et al. (2017) [79] Germany	*n* = 260 Type 2 DM = 55% 50.8 (±11.8)	CES‐D > 23	GET ON. Mood Enhancer Diabetes ‐ Internet guided self‐help intervention (*n* = 129) for depression versus CAU + online education on depression FU: 8 weeks, 6 months	*Depression:* HADS‐D: GET ON = 12.0(3.2); CAU = 11.7(3.7). *Diabetes*: HbA_1_c: GET ON = 7.6(1.6); CAU = 7.4(1.3).	*Depression:* CES‐D total mean score difference =−7.7 (*p*<.001). HADs total mean score difference = −3.2 (*p*<.001). *Diabetes:* HbA_1_c mean difference 0, n.s.	*Depression:* Δ0.735 *Diabetes*: Δ0.133	Significantly greater improvement in depressive symptoms in internet guided self‐help versus active control. No effect on glycemic control.	Sub	No		No
Newby et al. (2017) Australia	*n* = 90, Type 2 DM = 42%, 46.7 (±12.6)	PHQ9 scores 5=>23	Web‐based CBT versus CAU FU: 3 months (for iCBT group only)	*Depression*: PHQ9: iCBT = 15.95(5.25); TAU = 14.29(5.25). *Diabetes:* HbA_1_c: iCBT = 7.87(1.79); TAU = 7.72(1.82).	*Depression:* PHQ9 scores improved overall and the group x time interaction was significant (*p*<.001). 51% in iCBT versus 18% in TAU improved reliably. *Diabetes*: No significant interaction effect for HbA1c levels (*p*=.750).	*Depression:* Δ0.782 *Diabetes*: Δ 0.142	Significantly greater improvement in depressive symptoms but not glycemic control in Web‐based CBT group versus care as usual. No follow‐up data for care as usual group limits conclusions.	MDD	No		No
Group‐based interventions (4 RCTs, *n* = 508)											
Penckofer et al. (2012) USA	*n* = 74 Type 2 DM = 100% Female = 100% 54.0–54.8 (±8.4–8.8)	>16 CES‐D (indicating “significant” or mild depressive symptomatology ‐ average of 2 screenings)	SWEEP psychoeducational intervention versus CAU *FU*: 3 and 6 months.	*Depression*: CES‐D: SWEEP = 27.7(9.3); UC = 28.9(9.5). *Diabetes*: HbA_1_c: SWEEP = 7.8(1.8); UC = 7.9(2.0); FBG: SWEEP = 165.3 (71.1); UC = 168.8 (74.9).	*Depression*: CES‐D scores mean difference = ‐ 6.8 (*p*<.01). At 6 months 35% of intervention versus 80% of control remained depressed. *Diabetes*: No significant improvements for FBG or HbA1c levels.	*Depression:* Δ0.964 *Diabetes*: Δ0.272	Significant improvement in depressive symptoms but not glycemic control in SWEEP psychoeducation group compared to control group.	Sub	No		No
Hermanns et al. (2015) Germany	*n* = 214 Type 2 DM = 34.1% 43.3 (±13.3)	CES‐D > 16	Self‐management‐ orientated group program (DIAMOS) versus control group CAU + diabetes education. *FU*: 6 months, 12months	*Depression:* CES‐D: DIAMOS = 24.4(7.5); CG = 22.1(8.6); HADS: DIAMOS = 10.9(4.3); CG = 9.6(3.8). *Diabetes*: HbA_1_c: DIAMOS = 8.8(1.7); CG = 8.7(1.7).	*Depression*: CES‐D mean difference = −3.9 [95% CI 0.6–7.3] (*p* = .021) PHQ9 scores mean difference = −1.7 [95% CI 0.2–3.2] (*p* = .023). *Diabetes*: HbA_1_c levels mean difference=−0.3, *p*=.230)	*Depression:* Δ0.039 *Diabetes*: Δ0.269	Significant improvement in depressive symptoms but not glycemic control in DIAMOS group‐based therapy versus control group.	MDD	No		No
Long et al. (2015) China	*n* = 100 Type 2 DM = 100% 66.8 (±9.03)	SDS > 50	8 sessions of group counseling versus CAU *FU*: 3, 6 and 12 months.	*Depression*: SDS: GC = 0.57(0.067); CAU = 0.58(0.055). *Diabetes:* HbA_1_c: GC = 8.08(1.03); CAU = 8.10(1.10); FBG: GC = 9.26(1.70); CAU = 9.11(1.65).	*Depression*: SDS scores showed significant improvement (*p*<.001) *Diabetes:* FBG and HbA_1_C levels showed significant difference between groups (*p*<.05)	*Depression:* Δ1.637 *Diabetes*: Δ0.927	Improvement in depression scores, fasting blood glucose and glycemic control in group counseling versus usual care	MDD	No		No
Zheng et al. (2015) [92] China	*n* = 120 Type 2 DM = 100% 61–62 (±7–6)	Depression according to SDS	24 weeks Twenty‐four move Shadow Boxing and psychosomatic relaxation versus control group with CAU community diabetes health instructions. *FU*:24 weeks.	*Depression:* SDS: Boxing = 53.2(8.5); Control = 54.3(9.2). *Diabetes*: HbA_1_c: Boxing = 7.54(1.53); Control = 7.39(1.62).	*Depression:* SDS scores mean difference = −4.0 (*p*<.001). *Diabetes:* HbA_1_c levels mean difference = −0.36 (*p*=.016).	*Depression:* Δ0.610 *Diabetes:* Δ0.168	Significantly greater improvement in depressive symptoms and glycemic control in boxing intervention group versus control group.	Sub	Yes		No
Phone‐based (1 RCT, *n* = 225)											
Naik et al., (2019) USA	*n* = 225 =61.9 (±8.3)	PHQ 9 scores > 10	Telehealth collaborative goal setting and behavioral activation versus enhanced CAU *FU:* 6 and 12 months.	*Depression:* PHQ9: HOPE = 15.8(4.2); EUC = 16.2(4.0); *Diabetes:* HbA_1_c: HOPE = 9.2(1.4); EUC = 9.3(1.5).	*Depression*: PHQ9 scores mean difference =−2.14, (*p *= .03) *Diabetes*: HbA_1_c levels mean difference= −0.06% (*p*=.83) n.s.	*Depression:* Δ0.342 *Diabetes*: Δ−0.032	Significantly greater improvement in depressive symptoms but not glycemic control in HOPE telehealth intervention versus care as usual control group.	MDD	No		No
Light Therapy (1 RCT, *n* = 83)											
Brouwer et al. (2019)Netherlands	*n* = 83 Type 2 DM = 100% 60.1–62.9 (±9.8–10.7)	IDS scores > 14 MDD according to DSM‐IV criteria	Light therapy (active broad spectrum, white yellow light, 10,000 lux) versus placebo (monochromatic green light [545nm]) *FU*: 4, and 8 weeks	*Depression*: IDS: (values not reported). *Diabetes:* HbA_1_c: Light = 7.2(1.1); Placebo = 7.2(1.3).	*Depression:* IDS scores mean difference = −3.9 (*p* = .248) n.s. *Diabetes*: HbA_1_c levels mean difference = 1.9 (*p* = .116) n.s.	*Depression:* Δ 0.722 *Diabetes*: Δ −0.032	Light therapy was not significantly better at reducing depressive symptoms in comparison to placebo, and had no effect on glycemic control.	MDD	No		No

Note

The first column indicates the first author, year of publication and country study was conducted. The second column shows the sample size, % type 1 diabetes and type 2 diabetes and the Mean[*SD*] age of participants. The third column indicates how depressive disorder/presence of clinically significant symptoms or subthreshold disorder was diagnosed or defined. The fourth column describes the intervention, including the follow‐up (FU) time periods. Column 5 shows the Baseline data for both diabetes (e.g., HbA_1_c) and depression (e.g., depression questionnaire) outcomes. Column 6 shows the outcome data for both the diabetes and depression outcomes. Column 7 shows the effect size of the intervention on both the diabetes and depression outcomes. Column 8 describes the conclusions drawn from the study. Column 9 indicates whether the study focused on participants with depressive disorder or clinically significant symptoms (as noted by MDD) or subthreshold disorder (sub). Columns 10 and 11 show whether the intervention included an intervention component or focus on adherence, respectively. The number of trials and participants for each intervention is shown in the row indicating intervention type.

Abbreviations: BDI, beck depression inventory; CAU, care as usual; CBT, cognitive behavior therapy; CGI, clinical global impression; CVA, cerebro vascular accident; FBG, fasting blood glucose; HDRS, hamilton depression rating scale; MBCT, mindfulness‐based cognitive therapy; MDD, major depressive disorder; SDS, self‐rating depression dcale.

Overall meta‐analysis in the RCTs comparing all treatments with CAU, WL, or placebo for the combined effect on depressive outcome and glycemic control showed an effect size of 0.485; 95% CI 0.360; 0.609, *p* < .0001 (Appendix pp 10–12).

**Figure 2:**
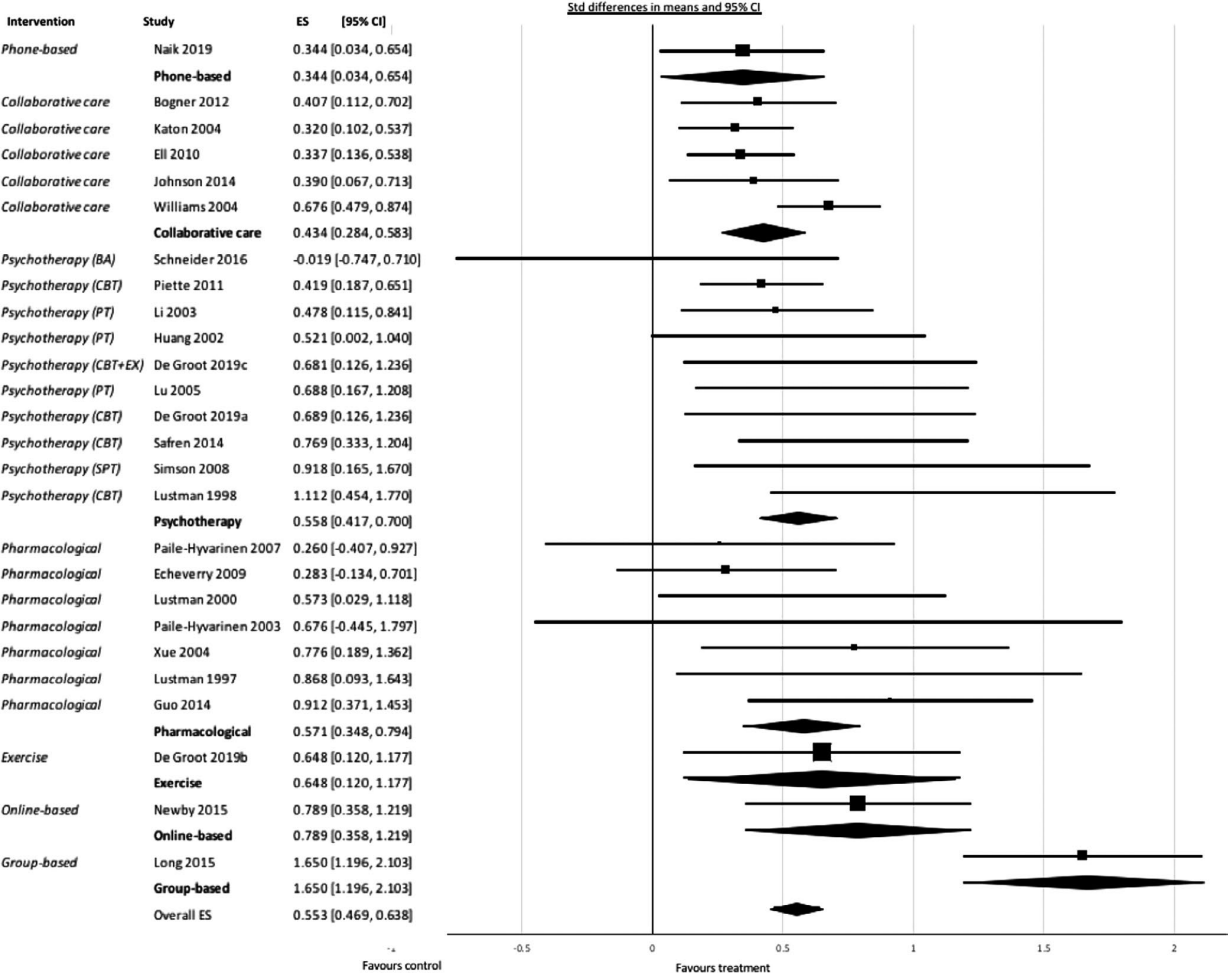
Forest plot showing results of meta‐analysis of studies of depressive disorder with depression as outcome, grouped by treatment. CBT = cognitive behavioral therapy; BA = behavioral activation; EX = add‐on exercise; PT = psychological treatment (counseling); SPT = supportive psychotherapy

Twenty‐four studies (Atlantis et al., 2014; Bogner et al., 2012; Echeverry et al., 2009; Groot et al., 2019; Guo et al., 2014; Huang et al., 2002; Johnson et al., 2014; Katon, Von Korff, et al., 2004; Li et al., 2003; Long et al., 2015; Lu Xs & Bx, 2005; Lustman, Freedland, et al., 2000; Lustman et al., 1998b; Naik et al., 2019; Newby et al., 2017; Paile‐Hyvärinen et al., 2003; Paile‐Hyvarinen et al., 2007; Piette et al., 2011; Safren et al., 2014; Schneider et al., 2016; Simson et al., 2008; Williams et al., 2004; Xue, 2004) examined patients with diabetes and depressive disorder, termed major depressive disorder (MDD). All treatments showed significant effects in terms of depression outcomes. Large effect sizes were found in group‐based therapy: effect size 1,650 (95% CI 1.196; 2.103), *p* = .0001; online treatment: effect size 0.789 (95% CI 0.358; 1.219), *p* = .0001; exercise: effect size 0.648 (95% CI 0.120; 1.177), *p* = .016; pharmacological treatment: effect size 0.571 (95% CI 0.348; 0.794) *p* = .0001, and psychotherapy: effect size 0.558 (95% CI 0.417; 0.700), *p* = .0001. Moderate effect sizes were found in collaborative care: effect size 0.434 (95% CI 0.284; 0.583), *p* < .0001; and phone treatment: effect size 0.344 (95% CI 0.034; 0.654), *p* = .030. The forest plot is shown in Figure 2 below.

**Figure 3:**
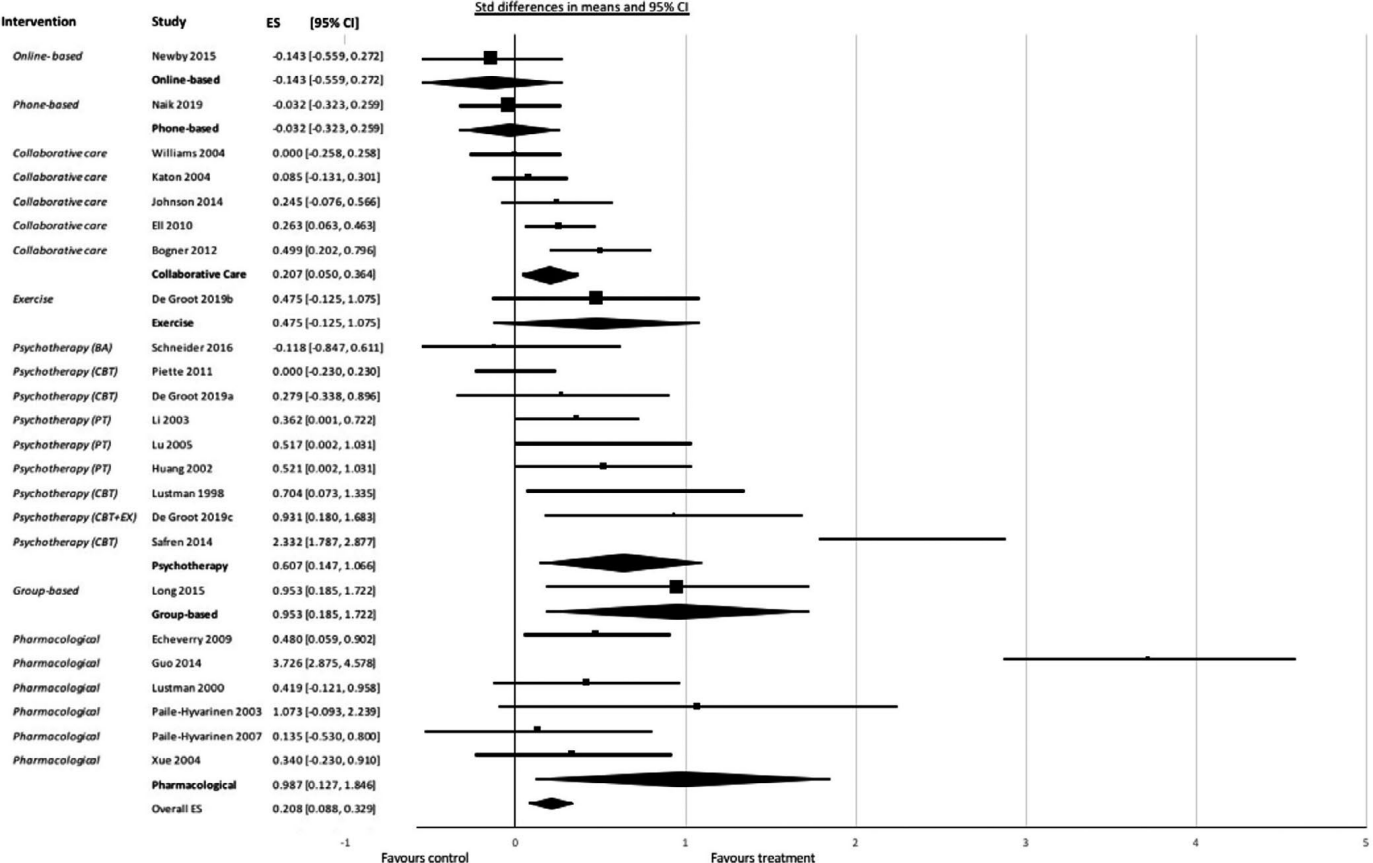
Forest plot showing results of meta‐analysis of studies of depressive disorder on glycemic control grouped by treatment. CBT = cognitive behavioral therapy; BA = behavioral activation; EX = add‐on exercise PT = psychological treatment (counseling)

Treatment showed a significant but small effect size in terms of glycemic control: 0.208 (95% CI 0.088; 0.329), *p* = .001. However, the effect size differed between treatment types: pharmacological treatment 0.987 (95% CI 0.127; 1.846), *p* = .024; group‐based therapy 0.953 (95% CI 0.185; 1.722), *p* = .015; psychotherapy 0.607 (95% CI 0.147; 1.066), *p* = .010; collaborative care 0.207 (95% CI 0.050; 0.364), *p* = .010. Effect sizes for exercise (*p* = .121) online treatment (*p* = .499) and phone treatment (*p* = .830) were not significant. The forest plot is shown in Figure 3.

Eight studies in patients with diabetes and subthreshold depressive symptoms (Ebert et al., 2017; Hermanns et al., 2015; Huang et al., 2016; Penckofer et al., 2012; Pibernik‐Okanovic et al., 2009, 2015; Tovote et al., 2014; Zheng et al., 2015) improved depression outcomes with an effect size of 0.360 (95% CI 0.204; 0.516), *p* < .0001 for all treatments. For psychotherapy: 1,131 (95% CI 0.083; 2.178), *p* = .034; and for online treatment 0.737 (95% CI 0.484; 0.990), *p* < .0001). Group therapy and psychoeducation had no significant effect. Glycemic control outcome effect sizes were significant for psychotherapy: 0.927 (95% CI 0.399; 1.455), *p* = .001 and group therapy: 0.237 (95% CI 0.019; 0.454), *p* = .033 (Appendix pp 13–14).

The meta‐regression analysis showed a significant association (slope 0.137; *p* < .0001) between baseline HbA_1_c and HbA_1_c as outcome but no association with depression as outcome. High baseline HbA_1_c was associated with a greater reduction in HbA_1_c. There was a significant association (slope of 0.023; *p* = .018) between severity of depression at baseline and depression as treatment outcome; and between severity of depression at baseline and glycemic control as outcome (slope 0.028; *p* = .005). High baseline depression score was associated with a greater reduction in HbA_1_c and depressive outcome (Appendix pp 15–18).

**Figure 4:**
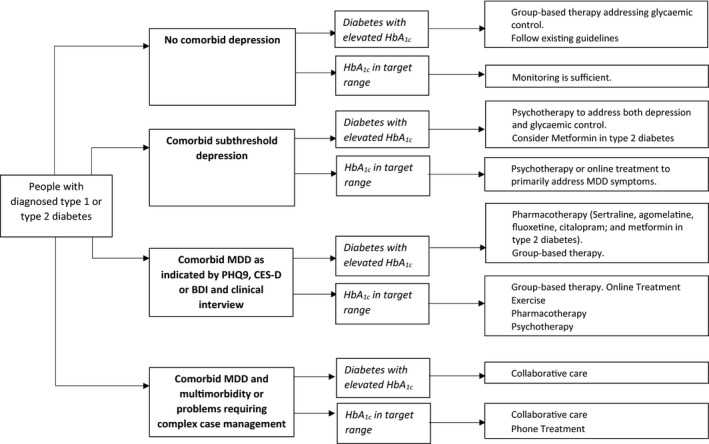
Flowchart showing treatment recommendations for comorbid depression in diabetes

*I* ^2^ values for the pooled outcomes were of moderate heterogeneity (Higgins et al., 2019) (69%) for all outcomes combined in all included studies. Based on the residuals, there were no outliers. This indicates that there is a distribution of intervention effects, as was expected as different interventions were compared. A fixed model meta‐analysis performed in all studies and outcomes as a meaningful test of the null hypothesis that there is no effect in every study (Higgins et al., 2019) showed *p*‐value < .0001 (Appendix pp. 19) indicating that the interventions were effective. Irrespective of the scales used and outcomes measured, consistent beneficial effects were seen for several treatments, suggesting the clinical meaningfulness of the outcomes of this systematic review and meta‐analysis. The findings from our meta‐analysis enable us to tentatively propose a flowchart to guide treatment choice, based upon the clinical profile of the patient and building on existing guidelines for treatment of people with diabetes. This flowchart is shown in Figure 4.

Among the 32 randomized controlled trials included in the meta‐analysis, most studies did not meet all criteria to reduce risk of bias, mostly due to unclear reporting about the method of randomization and blinding, instead the focus being on description of the intervention; unclear reporting about attrition rates and intention‐to‐treat (ITT) analysis. Furthermore, one study had low rates of compliance with the intervention under study and unclear reporting about the numbers of compliant participants (Brouwer et al., 2019); ten studies used small underpowered samples, despite otherwise being of seemingly adequate quality. Details of the risk‐of‐bias assessment for included trials are provided in the appendix (pp 4–7). A sensitivity analysis in the 16 studies (Baumeister & Bengel, 2012; Begg, 1994; Beydoun & Wang, 2010; Biostat, 2005; Bot et al., 2010; DerSimonian & Laird, 1986; Guyatt et al., 2008; Higgins, 2003; Higgins et al., 2019; Johnson et al., 2014; Nefs et al., 2012; Pibernik‐Okanović et al., 2011; Pibernik‐Okanovic et al., 2008; Rodríguez et al., 2012; Simson et al., 2008) with low risk of bias however showed a similar effect size: 0.402 (95% CI 0.271;0.533), *p* < .0001 on the combined measures outcome, compared with 0.485 in the original analysis. I^2^ was 47, which shows that focusing on low risk‐of‐bias studies provides similar results but reduces heterogeneity levels (Appendix pp. 20). A Begg funnel plot test for publication bias with observed and imputed studies showed no small study effect (Appendix pp 21).

## 6 DISCUSSION

This systematic review and meta‐analysis shows beneficial treatment effects for comorbid depression in type 1 and type 2 diabetes with a larger effect size (0.485) than in the original study that reported an effect size of 0.370 (95% CI 0.470; 0.271) (Van der Feltz‐Cornelis et al., 2010). This can be explained by the introduction of new interventions over the last decade with large effect sizes, such as group‐based therapy and online treatment. With the emergence of technological developments and increase in accessibility to the internet, treatments delivered online or using mobile technologies have increased in recent years. For example, many psychotherapies such as CBT can now be delivered online. This is particularly useful for people with diabetes given the propensity for poor health outcomes and high healthcare costs in this population.

Also, the effect size of collaborative care increased from a small to moderate effect size as this treatment model has developed over the last decade, especially in the domain of comorbid long‐term physical conditions and comorbid depression (O’Hagan & Boreham, 2013; Panagioti et al., 2016; Tully & Baumeister, 2015). There are large differences between treatment effects for different interventions in terms of diabetes and depression outcomes. All interventions improved depression outcomes significantly in depressive disorder with large effect sizes in group‐based therapy, online treatment, exercise, pharmacological treatment and psychotherapy, and moderate effect sizes in collaborative care and phone treatment. However, the effect sizes of such treatments for glycemic control were large in case of pharmacological treatment, group‐based therapy and psychotherapy, smaller for collaborative care, and not effective at all in case of exercise, online treatment and phone treatment. The finding that exercise was effective in terms of depression outcomes, but ineffective in improving glycemic control counters expectations for this intervention, as exercise is recommended as a treatment of both type 1 and type 2 diabetes. All current guidelines for depression and diabetes recommend exercise and other aspects of health lifestyle as a first step; this review and meta‐analysis, however, shows that exercise is only effective in improving depression. Exercise has been found to be an effective treatment for type 2 diabetes, helping to stabilize plasma glucose and improve body composition, insulin resistance, and glycated hemoglobin. Engagement in exercise is, however, suboptimal in people with diabetes (Koopmans et al., 2009), and this may be worse in case of comorbid depression (Katon et al., 2010; Lysy et al., 2008). As the findings in this meta‐analysis were only based on one study (Groot et al., 2019) on exercise, further research is needed. It would be of interest to assess what the additional effect of an exercise intervention embedded into treatment for diabetes and depression may be. This should be explored in further research as well as the effect of exercise as stand‐alone intervention.

This review also shows that interventions that are effective in depressive disorder may not be as effective in subthreshold depression. In this group, psychotherapy and online treatment had large, significant effect sizes on depressive symptoms, but group therapy and psychoeducation were not effective. Looking at glycemic control as an outcome, psychotherapy had a large, significant effect and group‐based therapy had a small, significant effect, while online treatment and psychoeducation had no significant effect at all. Consequently, the preferred treatment for both depression and glycemic control in comorbid subthreshold depression would be psychotherapy.

The finding that psychoeducation is not more effective than CAU in subthreshold depression, both for depression outcome and glycemic control, is an important finding as in stepped care models, psychoeducation has been suggested as a first step in diabetes‐related distress or subthreshold depressive symptoms (Huang et al., 2013). Furthermore, psychoeducation was supposed to be a good start for improving self‐management and in that way improving glycemic control. This line of thought is not supported by our results. Also, the finding that group therapy is highly effective in depressive disorder, but not in subthreshold depression, might suggest that patients with subthreshold depression might benefit more from individual treatment tailored to their specific needs rather than from group participation, something that has been suggested earlier (Huang et al., 2013). Treatment of comorbid subthreshold depressive disorder could be psychotherapy both in patients with elevated or normal HbA_1_c. The latter group might also benefit from online treatment. If glycemic control is a target, our analysis shows that it makes sense to target patients with high baseline levels of depression and of HbA_1_c, as they are likely to benefit most from treatment on both symptom levels.

In our flowchart, we recommend collaborative care in comorbid MDD and multimorbidity or problems requiring complex case management. Although effect sizes for some other treatment modes are found to be larger in our meta‐analysis, none of those were evaluated in patients with such a complex and multimorbid profile, whereas several systematic reviews show that outcomes in such patient groups improve by collaborative care (Faridhosseini et al., 2014; Tully & Baumeister, 2015).

One RCT (Guo et al., 2014) found that metformin improved glycemic control but also depressive outcomes, compared to placebo, in patients with type 2 diabetes. Although a small study with only 58 participants, this finding is of interest and may contribute to the expanding field of evaluation of medicines that are normally prescribed for physical conditions for their effect in treatment of depression (Arteaga‐Henríquez et al., 2019; Che et al., 2018; Köhler et al., 2014). Further research could explore the mechanism for metformin in improvement of depression in diabetes.

Our study has several strengths. First, we included data without language restriction from studies identified by a comprehensive search of the published literature. We included studies exploring the effect of treatment in subthreshold depression. Our sensitivity analysis excluding high risk‐of‐bias studies confirmed the findings, the fixed model meta‐analysis refuted the null hypothesis, and we found no indication for publication bias. Second, we provided relative effect sizes for several new treatment modalities compared to the treatments already explored in the first systematic review, we differentiated the treatment effect on depressive outcomes versus glycemic control, and by performing meta‐regression we showed the influence of baseline depression severity on both depression outcome and glycemic control, whereas baseline HbA1c only influenced glycemic control as an outcome. This combination of findings enabled us to provide clinicians with innovative guidance about which interventions may suit best, depending on patient profile. These strengths make our study the most comprehensive systematic review and meta‐analysis of treatment for comorbid depression in diabetes yet undertaken.

Our analysis has several limitations. First, most of the included studies did not meet all criteria to reduce risk of bias, mostly due to unclear reporting and to small samples. Despite our efforts to contact authors for missing data, we were unable to include such data in three studies due to lack of response (Ell et al., 2011; Petrak, Herpertz, et al., 2015; van der Sluijs et al., 2018), which may have to do with the long timeframe of this systematic review. The need for low risk‐of‐bias studies in this field remains, with proper reporting of methodology and of outcomes. Second, the planned moderator analyses on the effect of add‐on exercise on treatment outcome and on adherence as an outcome of treatment could not be performed because of insufficient data (Appendix pp. 22). Third, some treatments were only evaluated in one RCT. This probably reflects that, although many of these “new treatments” have been used for some time and have been felt to be useful by patients and clinicians, at least in primary care, researchers had not actively examined these “new” treatments until recently. In view of their clinical relevance, we emphasize this limitation. We strongly suggest further research is needed especially in group‐based treatment and exercise, that seem to have promising results. Another limitation concerns the provenance of the studies. Although this is a study with a global perspective in terms of included studies, it is clear that there is a scarcity of data from many low‐ and middle‐income countries, as shown by the map in Figure 1. The imbalance is of growing importance because it is likely that the low‐ and middle‐income countries will have the greatest increases of comorbidities of prevalence and incidence of diabetes and depression. In countries in which the attention to mental health problems is minimal or absent and the investment in the care for diabetes is appropriate, the guidance for treatment that we could deduce from this systematic review and meta‐analysis is particularly relevant and may improve care for comorbid depression. Furthermore, the studies in this meta‐analysis do not present results for type 1 diabetes and type 2 diabetes separately despite the different types of diabetes affecting different groups of the population; for example, type 2 diabetes tends develop more commonly in older people compared with a peak incidence of type 1 diabetes in adolescence and young adulthood. The lack of studies in type 1 diabetes alone with comorbid depression or comorbid subthreshold disorder is striking and research is needed to fill this gap.

A clearer understanding of the mechanisms underpinning why some treatments are more effective for patients with depressive disorder than for subthreshold depression and vice versa would also greatly benefit this area of research and for this purpose studies might provide more detailed information about the contents of the intervention. In particular, the idea that interventions aiming to improve self‐management lead to better adherence and better diabetes and depression outcomes should be challenged in research as studies reporting on adherence as an outcome are lacking. Studies are also needed to develop standardized techniques or tools to help better identify particular subtypes of patients taking into account their depression severity and glycemic control. These suggestions will further aid in the identification and personalization of appropriate treatment plans for patients with diabetes and depression as outlined above.

## CONFLICTS OF INTEREST

Over the last three years, RIGH has received honoraria for speaker engagement, conference attendance, or advisory boards from AstraZeneca, Boehringer Ingelheim, European Association for the Study of Diabetes, Eli Lilly, Janssen, Menarini, Mylan, Novo Nordisk, OmniaMed, and Otsuka. AN has received funding for lectures from OmniaMed, and, as Chairperson of the Psycho‐Social Aspects of Diabetes (PSAD) study group of the European Association for the Study of Diabetes, funding for travel Fellowships for early career researchers from Sanofi. The other authors have no conflicts of interest to declare.

## AUTHOR CONTRIBUTIONS

This systematic review was designed by CFC and co‐authors. Screening and data extraction were completed by SA and CFC. The meta‐analysis was performed by CFC. The initial version of the manuscript was written by SA and CFC. Following this, input was provided from all authors. All authors approved the final version of the manuscript.

## FUNDING INFORMATION

This study was financially supported by Hull York Medical School. The funder had no role in study design, data collection, data analysis, data interpretation, writing of the report, and decision to submit the paper for publication. The corresponding author had full access to all data in the study and had final responsibility for the decision to submit for publication.

## ETHICAL APPROVAL

Ethical approval was not required for the current study as the data entered in the meta‐analysis were collated from previous clinical trials in which informed consent had already been obtained.

## Supporting information

Supplementary Material

## Data Availability

Data available on request.
