# Supplementary material for: Treatment for comorbid depressive disorder or subthreshold depression in diabetes mellitus: Systematic review and meta‐analysis
Source: Brain Behav. 2020 Dec 4;11(2):e01981. doi: 10.1002/brb3.1981 (PMC7882189; doi:10.1002/brb3.1981)
Supplement: Supplementary file 1 — Supplementary Material [file BRB3-11-e01981-s001.docx]

**Appendix**

**Search strategy**

*PubMed*

#1 Diabetes:“Diabetes Mellitus” [Mesh] OR diabet⁎ [tiab]

#2 Depression:“Depression”[Mesh] OR “Affective Symptoms”[Mesh] OR “Depressive Disorder”[Mesh: NoExp] OR “Depressive Disorder, Major”[Mesh] OR “Dysthymic Disorder”[Mesh] OR “Mood Disorders”[Mesh: NoExp] OR depress⁎[tiab] OR affective symptom⁎ [tiab] OR dysthymi⁎ [tiab] OR affective disorder⁎ [tiab] OR mood disorder⁎ [tiab]

#3 RCT filter: (Clinical trial[pt] OR Randomized Controlled Trial[pt] OR randomiz⁎[tiab] OR randomis⁎ [tiab] OR placebo[tiab] OR clinical trials[mh] OR randomly[tiab] OR trial[ti]) NOT (animals[mh] NOT (animals[mh] AND humans[mh]))

#4 Systematic review filter

(“meta-analysis”[pt]) OR (meta-anal⁎[tw]) OR (metaanal⁎[tw]) OR (quantitativ⁎[tw] AND review⁎[tw]) OR (quantitative⁎[tw] AND overview⁎[tw]) OR (systematic⁎[tw] AND review⁎[tw]) OR (systematic⁎[tw] AND overview⁎[tw]) OR (methodologic⁎[tw] AND review⁎[tw]) OR (methodologic⁎[tw] AND overview⁎[tw]) OR (“review”[pt] AND “medline”[tw])

#1 AND #2 AND (#3 OR #4)

*PsycINFO*

#1 Diabetes: DE=“diabetes” OR DE=“Diabetes mellitus” OR KW=diabet⁎

#2 Depression: DE=“Depression (Emotion)” OR DE=“Major Depression” OR DE=“Dysthymic Disorder” OR DE=“Affective Disorders” OR DE=“Endogenous Depression” OR DE=“Reactive Depression” OR DE=“Recurrent Depression” OR DE=“Treatment Resistant Depression” OR KW=Mood Disorder⁎ OR KW=Dysthymi⁎ OR KW=depress⁎ OR KW=affective⁎ disorder⁎

#3 RCT filter: (ME=(treatment outcome/clinical trial) or KW=clinical trial⁎ or KW=clinical trial⁎ or KW=randomiz⁎ or KW=randomis⁎ or KW=placebo or KW=randomly or TI=trial) not (PO=animal not (PO=animal and PO=human))

#4 Systematic review filter: (ME=Systematic review) or (ME=meta analysis) or (KW=meta-anal⁎) or (KW=metaanal⁎) or (KW=quantitative⁎ review⁎) or (KW=quantitative⁎ overview⁎) or (KW=systematic⁎ review⁎) or (KW=systematic⁎ overview⁎) or (KW=methodologic⁎ review⁎) or (KW=methodologic⁎ overview⁎) or (KW=review AND KW=medline)

#1 AND #2 AND (#3 OR #4)

*Cochrane*

#1 Diabetes: “diabet⁎ in Title, Abstract or Keywords

#2 Depression: “depress⁎ in Title, Abstract or Keywords or affective symptom⁎ in Title, Abstract or Keywords or dysthymi⁎ in Title, Abstract or Keywords or affective disorder⁎ in Title, Abstract or Keywords or mood disorder⁎ in Title, Abstract or Keywords

#1 AND #2

*EMBASE*

#1 Diabetes: ‘diabetes mellitus’/exp OR diabet⁎: ti,ab,de

#2 Depression : ‘depression’/exp OR depress⁎: ti,ab,de OR ‘affective symptom’: ti,ab,de OR ‘affective symptoms’: ti,ab,de OR ‘affective symptomatology’: ti,ab,de OR ‘affective symptomatic’: ti,ab,de OR dysthymi⁎: ti,ab,de OR ‘affective disorder’: ti,ab,de OR ‘affective disorders’: ti,ab,de OR ‘Mood disorder’: ti,ab,de OR ‘Mood disorders’: ti,ab,de

#3 RCT filter: ‘Clinical Trial’: it OR [Controlled Clinical Trial]/lim OR ‘Randomized Controlled Trial’: it OR [Randomized Controlled Trial]/lim OR randomiz⁎: ti,ab,de OR randomis⁎: ti,ab,de OR placebo: ti,ab,de OR ‘clinical trial’/exp OR randomly: ti,ab,de OR trial: ti NOT ([animals]/lim NOT ([animals]/lim AND [humans]/lim))

#4 Systematic review filter: [meta analysis]/lim OR [systematic review]/lim OR [Cochrane review]/lim OR meta⁎anal⁎ OR (quantitative⁎ AND review⁎) OR (quantitative⁎ AND overview⁎) OR (systematic⁎ AND review⁎) OR (systematic⁎ AND overview⁎) OR (methodologic⁎ AND review⁎) OR (methodologic⁎ AND overview⁎) OR (‘Review’: it AND medline)

#1 AND #2 AND (#3 OR #4) AND [embase]/lim

**Glossary of Interventions**

*Antidepressants* = Medicine for treating clinical depression (e.g. sertraline, fluoxetine)

*Antidiabetes* = Medicine used to treat diabetes by lowering the glucose level in the blood (e.g. metformin).

*Behavioural Activation* = is a third-generation behaviour therapy for treating depression, based on a model of applied behaviour analysis, which encourages patients to approach activities they may have been avoiding.

Cognitive Behavioural Therapy (CBT)= A psychological intervention that focuses on challenging and changing unhelpful thoughts, beliefs, attitudes and behaviours, improving emotional regulation, and the development of coping strategies.

*Collaborative Care* = A model of health care in which multiple health care providers from different disciplines work together providing comprehensive services to deliver the highest quality of care across settings.

*Counselling* = an individual or group-based talking therapy which aims to help with a range of mental and emotional problems, including stress, anxiety and depression.

*Exercise Intervention* = A behavioural intervention including an exercise-based component.

*Interpersonal Psychotherapy* *(IPT)* = a brief, attachment-focused psychological therapy that focuses on resolving interpersonal problems and symptomatic recovery.

*Manualized CBT* = CBT delivered by therapists in which specific technique-driven treatments are used for individual psychological disorders, and are often brief, direct, and time-limited.

*Mindfulness-based Cognitive Therapy (MBCT)* = an approach to psychotherapy that uses both cognitive behavioural therapy methods and mindfulness meditation practices in addition to other similar psychological strategies

*Mindfulness-based Stress Reduction Therapy (MSRT)* = An evidenced-based program offering secular, intensive mindfulness training that aims to help people deal with stress, anxiety, and depression.

*Pharmacotherapy* = An intervention comprising of a pharmacological treatment for treating a physical or mental health condition (e.g. sertraline, escitalopram, metformin)

*Problem-solving Treatment* = A form of therapy which aims to provide patients with the tools to identify and solve problems that arise from life stressors.

*Psychoeducation* = An evidence-based therapeutic intervention that provides people with information and support to better understand and cope with a mental or physical illness.

*Psychotherapy* = An intervention comprising of a structured psychological therapy for treating people with mental health and emotional problems such as depression and anxiety.

*Stepped Care* = A model of treatment delivery in which the most effective yet least resource intensive, treatment is delivered to patients in the first instance, before more intensive or specialist treatments, as clinically required.

*Supportive Psychotherapy* = A psychotherapeutic approach that integrates various schools such as psychodynamic, cognitive-behavioural, and interpersonal conceptual models and technique.

**List of Acronyms**

BDI = Beck Depression Inventory

CAU = Care as Usual

CBT = Cognitive Behavioural Therapy

CET = Comparative Effectiveness Trial

CCMD= Chinese classification of Mental Disorders

CES-D = Center for Epidemiologic Studies Depression Scale

DSM = Diagnostic and Statistical Manual of Mental Disorders

FBG = Fasting Blood Glucose

FPG = Fasting Plasma Glucose

HADs = Hospital Anxiety and Depression Scale

HAMD/HDRS = Hamilton Depression Scale

HbA**_1c_** = Haemoglobin A1c

iCBT = Online-based Cognitive Behavioural Therapy

IPT = Interpersonal Psychotherapy

MADRS = Montgomery and Asberg Depression Rating Scale

MBSRT = Mindfulness-based Stress Reduction Therapy

MBCT – Mindfulness-based Cognitive Therapy

MINI = The Mini-international Neuropsychiatric Interview

MDD = Major Depressive disorder

PHQ = Patient Health Questionnaire

RCT = Randomized Controlled Trial

SCID = Structured Clinical Interview for DSM-IV

SDS = Self-rating Depression Scale

SRS = Zung Self Rated Scale

**Flowchart**


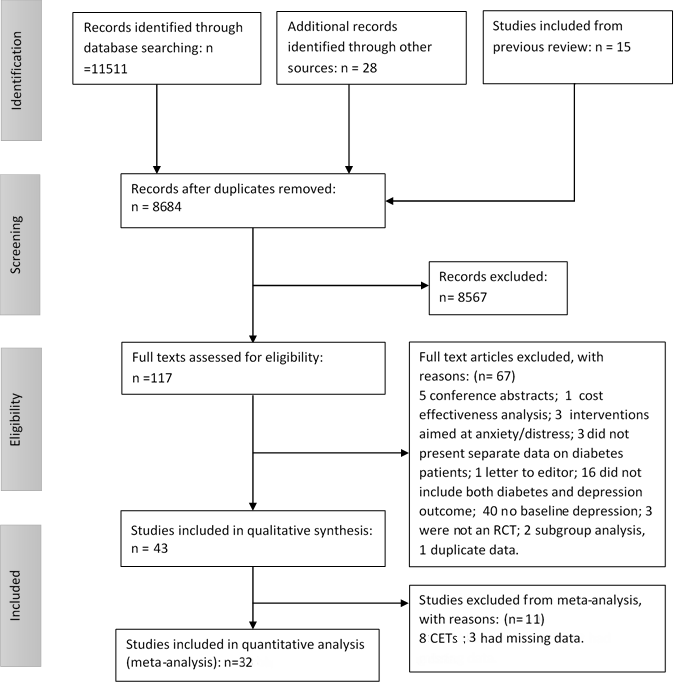


**Table Risk of Bias assessment**

| Author (year) | 1. Sequence generation | 2. Allocation Concealment | 3. Blind Outcome Assessor | 4. Blind Other | 5. Reporting of loss to follow-up/ITT analysis | 6. Selective outcome reporting | 7. Other Biases | Overall Comment | Score (out of 7) *= good quality | GRADE |
| --- | --- | --- | --- | --- | --- | --- | --- | --- | --- | --- |
| Lustman et al. (1998) [19] | Yes | Yes | Yes | No | Yes | Unclear | No | Good quality study. | 5.5* | High |
| Huang et al. (2002) [20] | Unclear | Unclear | Unclear | No | Unclear | Yes | No | Intervention is well described, methods only concisely. Analysis difficult to interpret. Age not reported. | 4 | Moderate |
| Li et al. (2003) [21] | Unclear | Unclear | Unclear | No | Unclear | Yes | Yes | Very concise description of methods. | 3 | Moderate |
| Lu et al. (2005)[22] | Unclear | Unclear | Unclear | No | Unclear | Yes | Yes | Methods are only concisely described and therefore the analysis is difficult to interpret. | 3 | Moderate |
| Simson et al. (2008) [23] | Unclear | Unclear | Unclear | No | Yes | Yes | Yes | Intervention and outcome are well described, methods only concisely. Study is very small. | 2.5 | Low |
| Piette et al. (2011) [56] | Unclear | Yes | Unclear | Yes | Unclear | Unclear | Unclear | Adequate quality study | 4.5 | Moderate |
| Safren et al. (2014) [57] | Unclear | Unclear | Yes | Yes | Yes | Unclear | Yes | Quite small predominantly white sample so lacks ability to generalise. | 4.5 | Moderate |
| Tovote (2014) [59] | Yes | Unclear | Yes | No | Yes | Unclear | Yes | Under powered sample. Clinical interviewer post treatment not blinded. | 4 | Moderate |
| Schneider et al. (2016) [58] | Yes | Yes | Yes | Unclear | Yes | Unclear | Yes | Small sample mainly white non-Hispanic so can't generalise. | 5* | High |
| Huang (2016) [50] | Yes | Yes | Yes | Unclear | Yes | Unclear | No | Small sample but overall good quality. | 6* | High |
| De Groot et al., (2019) [46] | Yes | Yes | Yes | Yes | Yes | Unclear | Yes | No baseline or follow up data reported but otherwise a good quality study. | 5.5* | High |
| Gülseren et al. (2005) [33] | Unclear | Unclear | Yes | Unclear | Yes | Yes | Yes | Probably a good study, methods are rather concise. This study compares two active treatment conditions. | 3.5 | Moderate |
| Barragan-Rodriguez (2008) [35] | Yes | Unclear | Unclear | Unclear | Yes | Unclear | Yes | ITT performed on patients who completed follow up only, and small sample size. | 4 | Moderate |
| Khazaie (2011) [36] | Yes | Unclear | Yes | Unclear | No | Unclear | Yes | Small sample and unequal allocation between groups. | 3.5 | Moderate |
| Karaiskos (2013) [71] | Unclear | Unclear | No | No | Yes | Unclear | Yes | Open label study with no control group and small sample size. | 2.5 | Low |
| Kang (2015)[37] | Yes | Unclear | Yes | Unclear | Yes | Unclear | Yes | No control group so lacks generalisability. | 4.5 | Moderate |
| Kumar (2015) [38] | Yes | Unclear | No | Unclear | No | Unclear | Yes | Open label study and small sample size. | 3 | Moderate |
| Gois (2014)[39] | Yes | Unclear | Unclear | Unclear | No | Unclear | Yes | ITT not performed: analysis for early-responding patients conducted separately. | 3 | Moderate |
| Petrak (2015) [40] | Yes | Yes | Yes | No | Yes | Unclear | Yes | Non responders not included in 15 month follow-up. | 4.5 | Moderate |
| Lustman (1997) [24] | Unclear | Unclear | Yes | Yes | Unclear | Unclear | No | Control group of undepressed patients contained many anxiety disorders. Unclear how dropouts were represented in the groups. | 4 | Moderate |
| Lustman et al. (2000) [25] | Yes | Yes | Yes | Yes | Yes | Yes | Yes | Good quality study. | 5* | High |
| Paile-Hyvarinen (2003) [26] | Yes | Yes | Yes | Yes | Yes | Yes | Yes | Good quality study, however very small N: 13 | 5* | High |
| Xue (2004)[27] | Unclear | Unclear | Yes | Yes | Yes | Unclear | Unclear | Probably a good study, but the methods are rather concisely described. | 5* | High |
| Paile-Hyvarinen (2007) [28] | Yes | Yes | Yes | Yes | No | Yes | Yes | Misbalance between groups: high dropout in placebo group. Underpowered study. Ceiling effect. | 4 | Moderate |
| Echeverry (2009) [32] | Yes | Yes | Yes | Yes | Yes | Yes | Yes | Good quality study. | 5* | High |
| Guo (2014) [48] | Yes | Unclear | Yes | Yes | No | Unclear | Yes | Drops outs reported but not by group allocated. Small sample. | 4 | Moderate |
| Pibernik- Okanovic (2009) [61] | Yes | Yes | Unclear | Unclear | Unclear | Unclear | Yes | Small underpowered sample. | 4 | Moderate |
| Pibernik- Okanovic (2015) [55] | Yes | Unclear | No | No | Yes | Yes | Yes | Group allocation was not blinded and there was no control group. | 2.5 | Low |
| Katon (2004) [29] | Yes | Yes | Yes | No | Yes | Yes | Yes | Good quality study | 5* | Moderate |
| Williams (2004)  [30] | Yes | Yes | Yes | Yes | Yes | Yes | Yes | Good quality study. | 5* | High |
| Ell (2010) [31] | Yes | No | Unclear | No | Unclear | No | Yes | Probably a good study, but methods concisely described. Focus on Hispanics with baseline HbA**_1c_** >8 limits generalizability. | 3 | Moderate |
| Ell (2011) [45] | Yes | Yes | Yes | Yes | Yes | Unclear | Yes | Predominantly Hispanic patients so low generalisability. Otherwise good quality. | 5.5* | High |
| Bogner (2012) [45] | Unclear | Unclear | Yes | No | Yes | Unclear | Yes | Undertaken in only 3 primary care facilities so low generalisability. | 3 | Moderate |
| Johnson (2014) [51] | Yes | Yes | Yes | No | Yes | Unclear | Unclear | Adequate quality study | 4.5 | Moderate |
| Van Bastelaar (2011) [43] | Yes | Yes | Yes | No | Yes | Unclear | Yes | High attrition and insufficient randomised sample size. Not all data reported in paper. | 4.5 | Moderate |
| Ebert (2016) [47] | Yes | Yes | Yes | Unclear | Yes | Unclear | Yes | Participants recruited via German health insurance company so potential source of bias. Otherwise good quality. | 5* | High |
| Newby (2017) [53] | Yes | Yes | Unclear | Yes | Yes | Unclear | Unclear | Good quality study | 5.5* | High |
| Penckofer (2012) [54] | Yes | Unclear | No | No | Yes | Unclear | Yes | Randomisation stratified by depression severity. Blinding did not occur. | 3 | Low |
| Hermanns (2015) [49] | Yes | Unclear | Yes | Yes | Yes | Unclear | Yes | Significantly higher baseline depression in intervention group. Otherwise good quality study. | 5* | High |
| Long (2015) [44] | Unclear | Unclear | Unclear | Unclear | Unclear | Unclear | Yes | Methods very briefly described. Non-generalizable community sample. | 3 | Moderate |
| Zheng (2015) [60] | Unclear | Unclear | Unclear | Unclear | Unclear | Unclear | Yes | Recovered patients were not included in analysis, otherwise no loss to follow up reported. Potential high bias. | 3 | Moderate |
| Naik (2019) [52] | Yes | Yes | Yes | Yes | Yes | Unclear | Unclear | Good quality study. Unclear why unequal allocation was used. | 6* | High |
| Brouwer (2019) [42] | Yes | Yes | Yes | No | Yes | Unclear | Yes | No baseline or follow up data reported in paper. Otherwise of adequate quality. | 4.5 | Moderate |

Legend: Studies were allocated a point for each ‘yes’ on the first five criteria, a point for each ‘no’ on the last two criteria; and half a point for each ‘unclear’. Total scores are shown with those scoring 5 points and above deemed to be methodologically sound. GRADE scores indicate the subjective appraisal of confidence in the reported effect size.

**Forest plot showing results of meta-analysis of illness burden**


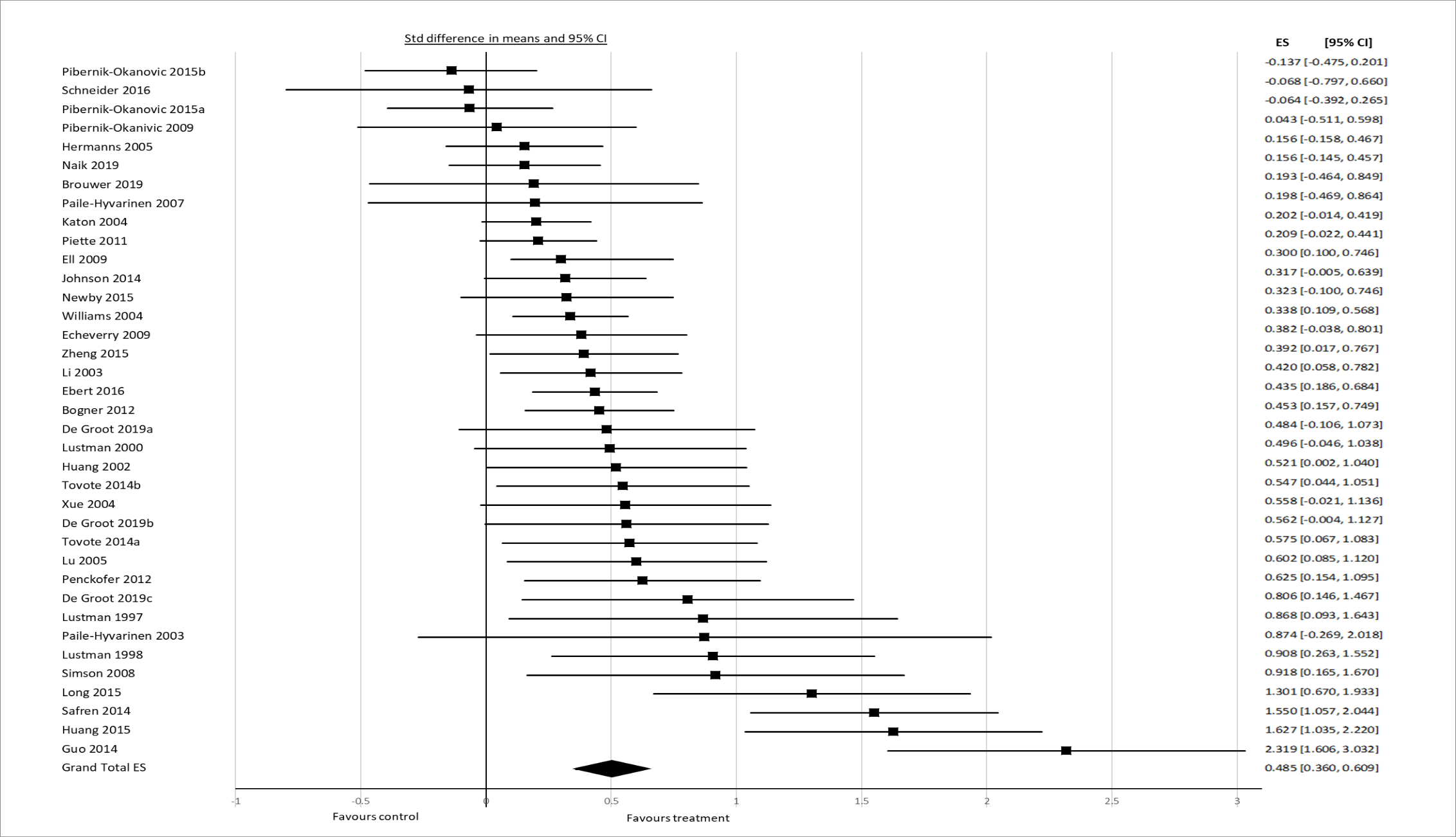


**Illness burden analysis**

Effect sizes were calculated, as well as pooled effect sizes. As in the original meta-analysis[1], we used a weighted mean indicating a combined assessment of illness burden. This outcome establishes the impact of the intervention at hand on the general clinical condition of the patients. The clinical meaning of this is envisioned to be somewhat similar to the Clinical Global Impression[2, 3], an assessment that a clinician would make in order to indicate if the general clinical condition of the participant improved in terms of depressive symptoms combined with glycaemic control. For this, we used the two outcomes mentioned above and translated those continuous measures to a standardized pooled combined equally weighted effect size with a statistical program that corrects for possible inflated weights in case of multiple outcomes by equal weighting[4]. The diabetes and depression outcomes were given similar weights in the weighted means calculation. The methodology for this is described more extensively in van der Feltz-Cornelis et al.[1]

We also performed an analysis of illness burden in the MDD studies versus the subthreshold studies. The effect size in MDD studies was 0.550; 95% CI 0.399; 0.703, p<0.0001. The effect size in subthreshold studies was 0.381; 95% CI 0.127; 0.635; p= 0.003. Although there was a numerical difference in effect size for the two severity levels of depression, as can be seen in the forest plot below, the confidence intervals overlap, and both effect sizes are in the moderate range. The Q value for this model is 0.987 df=1, p=0.320, indicating that there is no significant dispersion. I^2^ is 69 for the whole group.


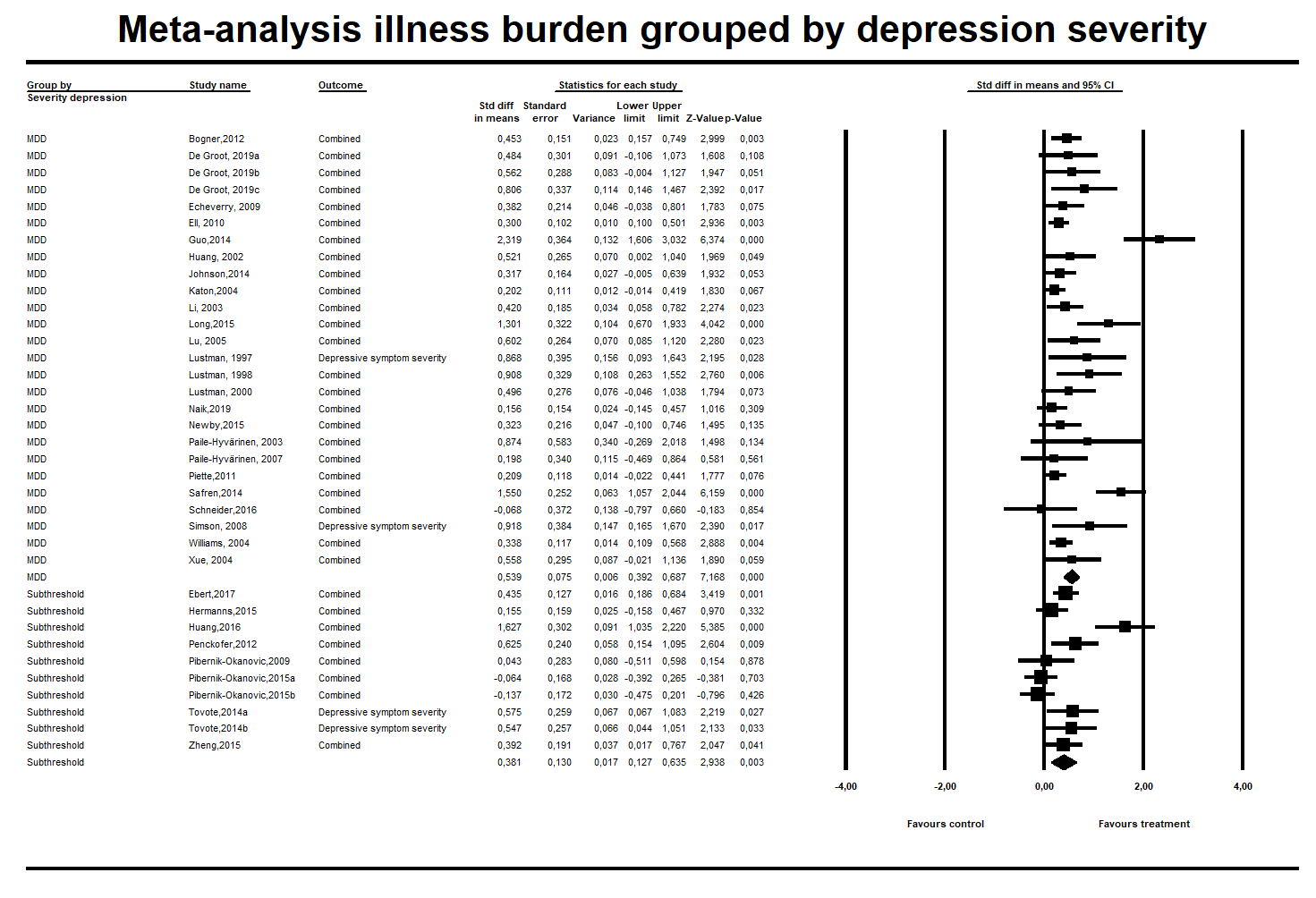


F**orest plot showing results of meta-analysis of subthreshold depression studies on depression outcome grouped by treatment**


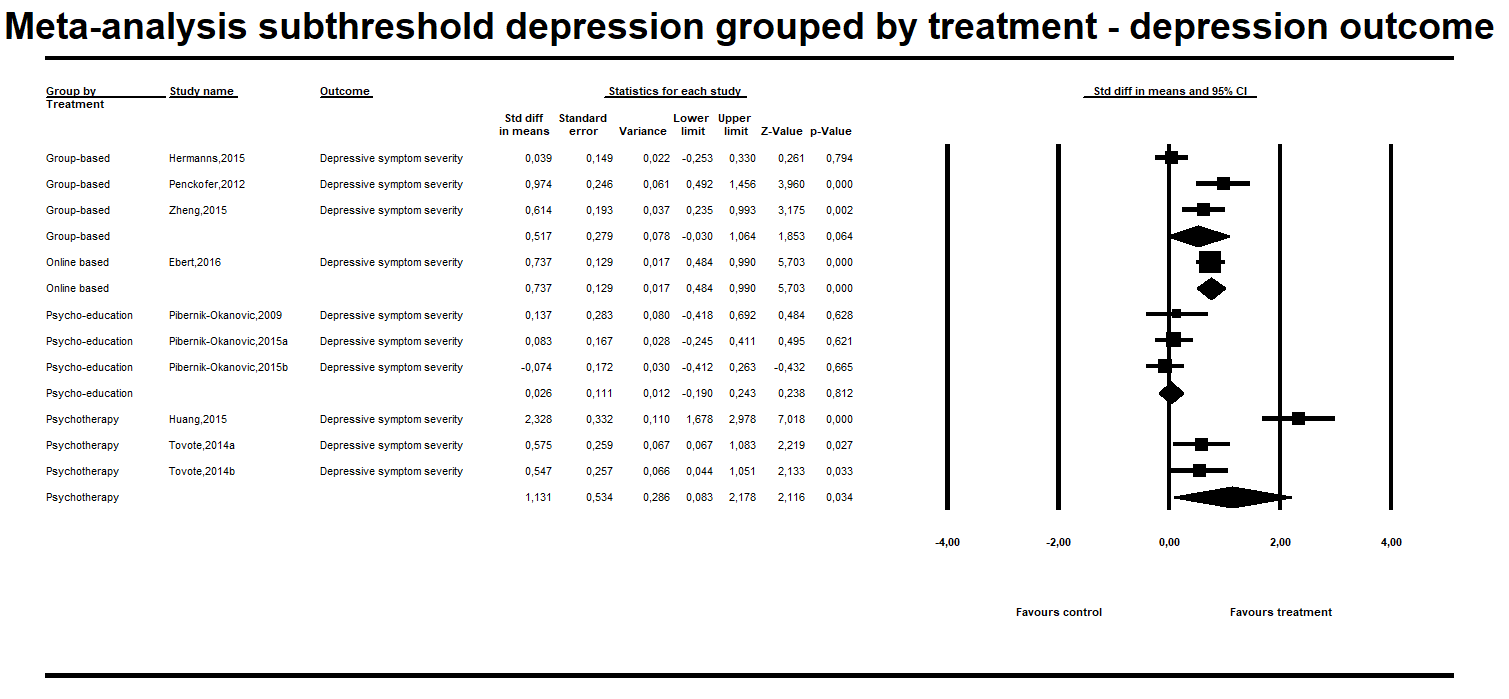


**Forest plot showing results of meta-analysis of subthreshold depression studies on glycemic control outcome grouped by treatment**


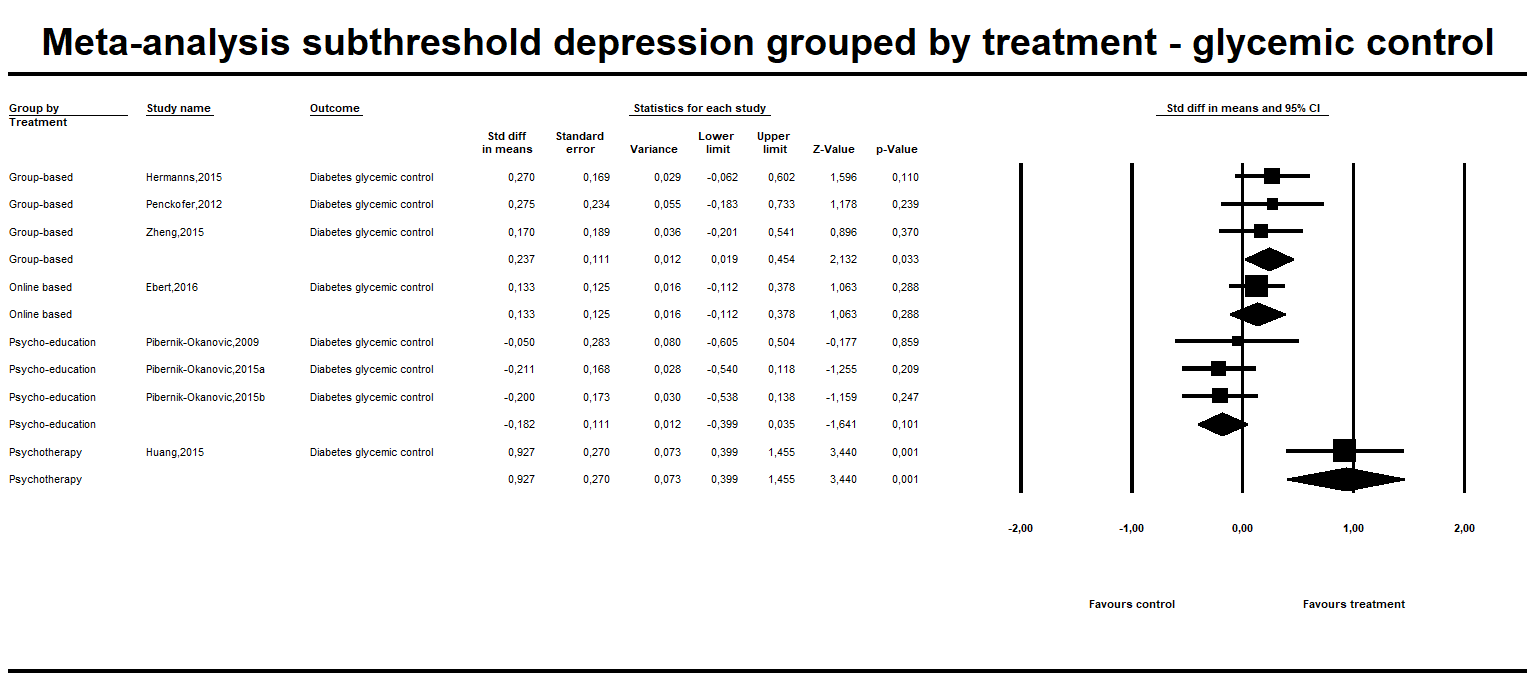


**Meta-regression**

A meta-regression was conducted to assess whether baseline levels of depressive severity (scores on depression questionnaires) or glycaemic control (HbA_1c_) influenced the effect of the intervention. As the studies used various tools for their outcome measure of depression severity, for the scores to be entered into the meta-regression they required standardisation. The BDI was the most frequently used and the scores are easy to interpret. It was decided to convert scores to the BDI scale where possible. Using the equations outlined by Hawley[5], MADRS, SRS and PHQ-9 scores were converted directly into BDI scores. CES-D scores were also converted in BDI scores, using the conversion table published in Choi, et al.[6] whereas HAM-D scores were first converted into MADRS scores using the conversion table published by Leucht, et al.[7] and then into BDI scores using the equations in the Hawley paper. BDI scores were then entered into the meta-regression.

First, we ran a meta-regression analysis to explore the association between baseline HbA_1c_ and treatment outcomes. First the meta-regression was done for HbA_1c_ as outcome. The scatterplot shows a slope of 0.137 and p< 0.0001. This is a small but significant association.

A similar meta-regression run for depression as outcome showed no significant association with baseline HbA_1c_. (p-value 0,68371)

Then we ran a meta-regression analysis to explore the association between baseline depression severity and treatment outcomes. First the meta-regression was done for depression outcome. With a slope of 0.023 and p=0.018, there is a very small but significant association between severity of depression at baseline and treatment outcome for depression. A similar meta-regression run for glycaemic control as outcome showed a slope of 0.028 and p= 0.005, indicating a similar, small but significant association between severity of depression at baseline and treatment outcome for glycaemic control.

**Scatterplot showing results of meta-regression of baseline** **HbA_1c_ levels on glycemic control as outcome. High baseline HbA_1_c is associated with a greater reduction in HbA_1_c.**


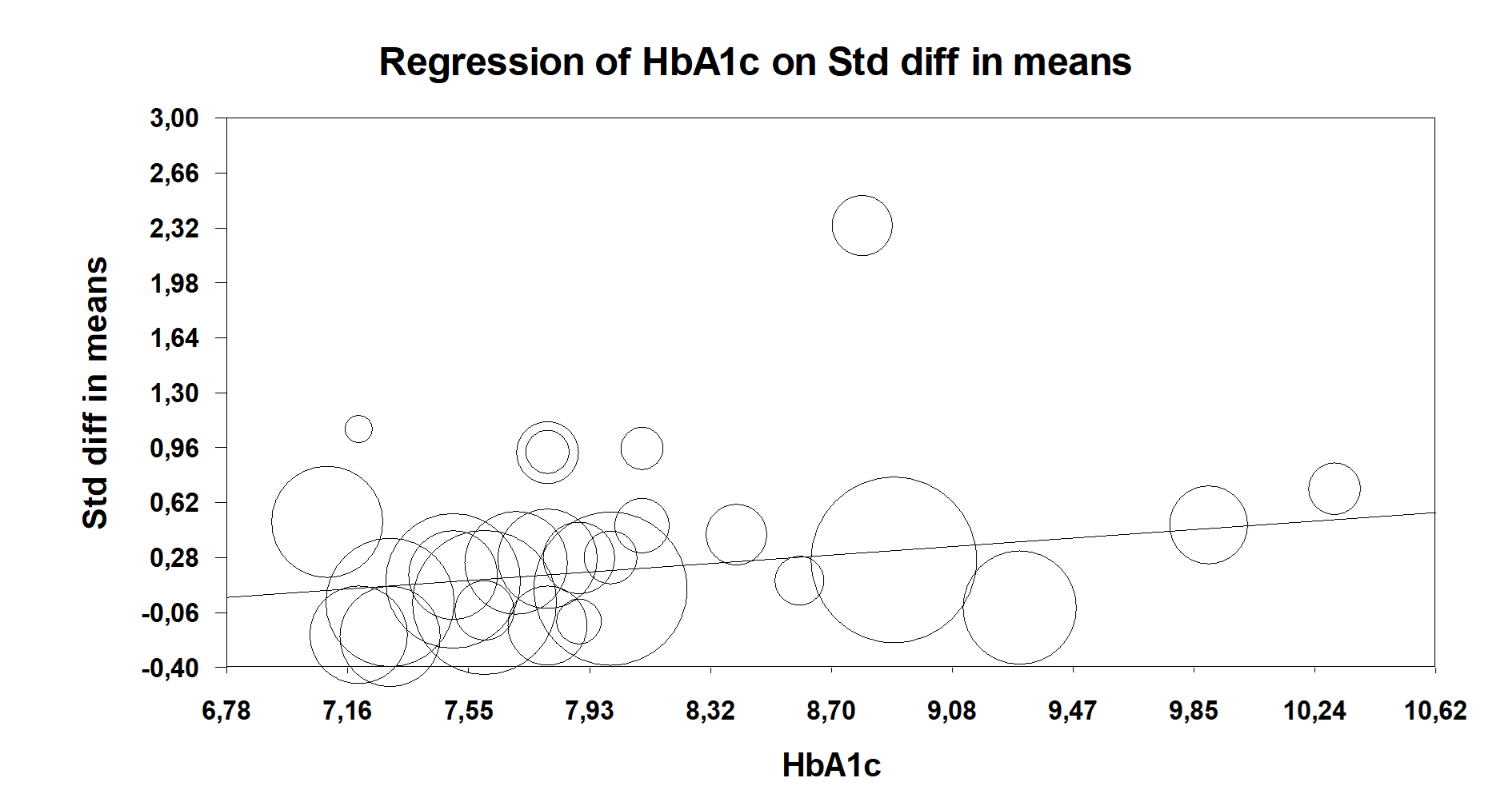


**HbA1c**

**Scatterplot showing results of meta-regression of baseline depression levels on depression outcome. High baseline depression score is associated with a greater reduction** **in depressive outcome.**


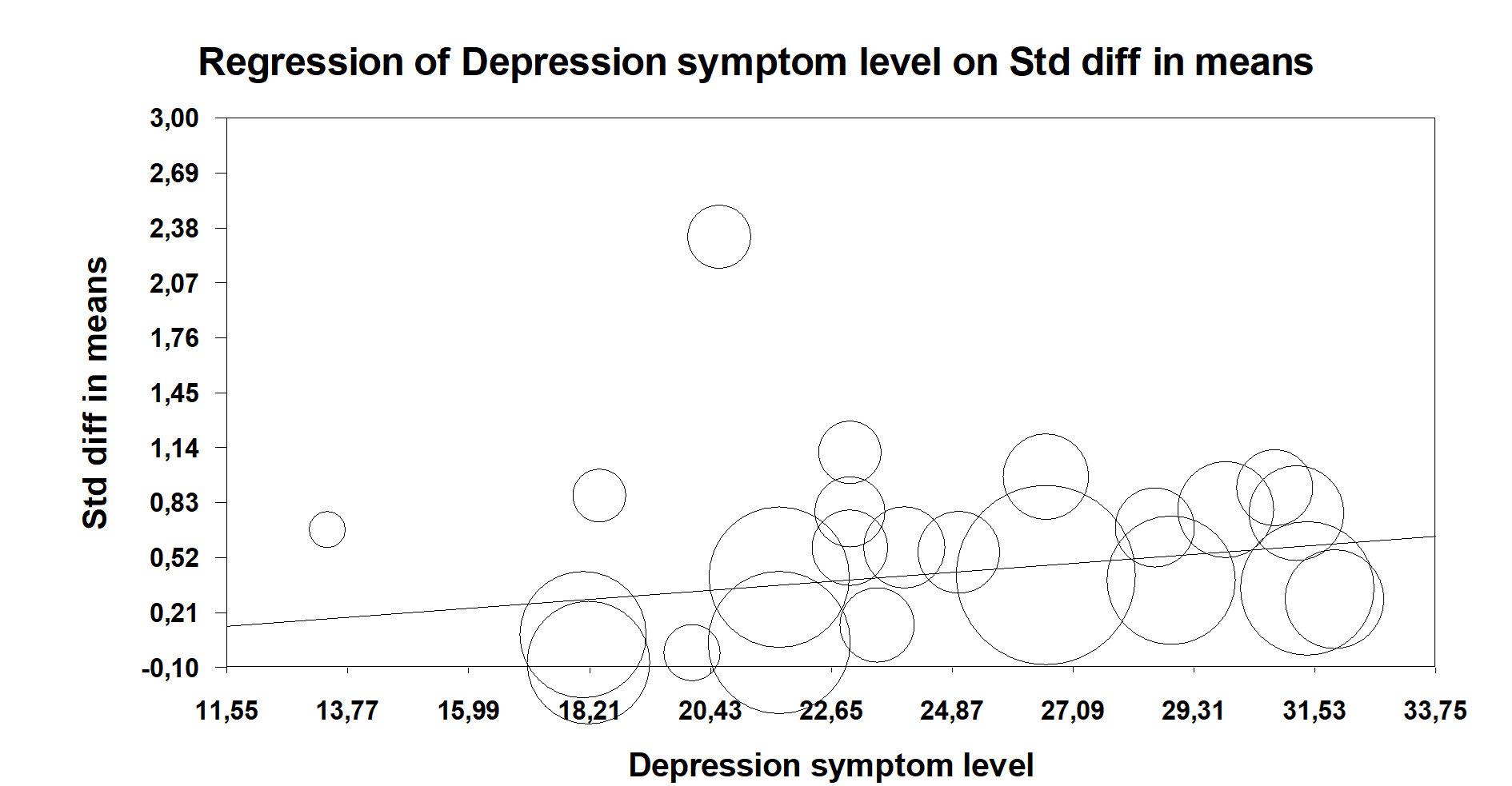


**Depression**


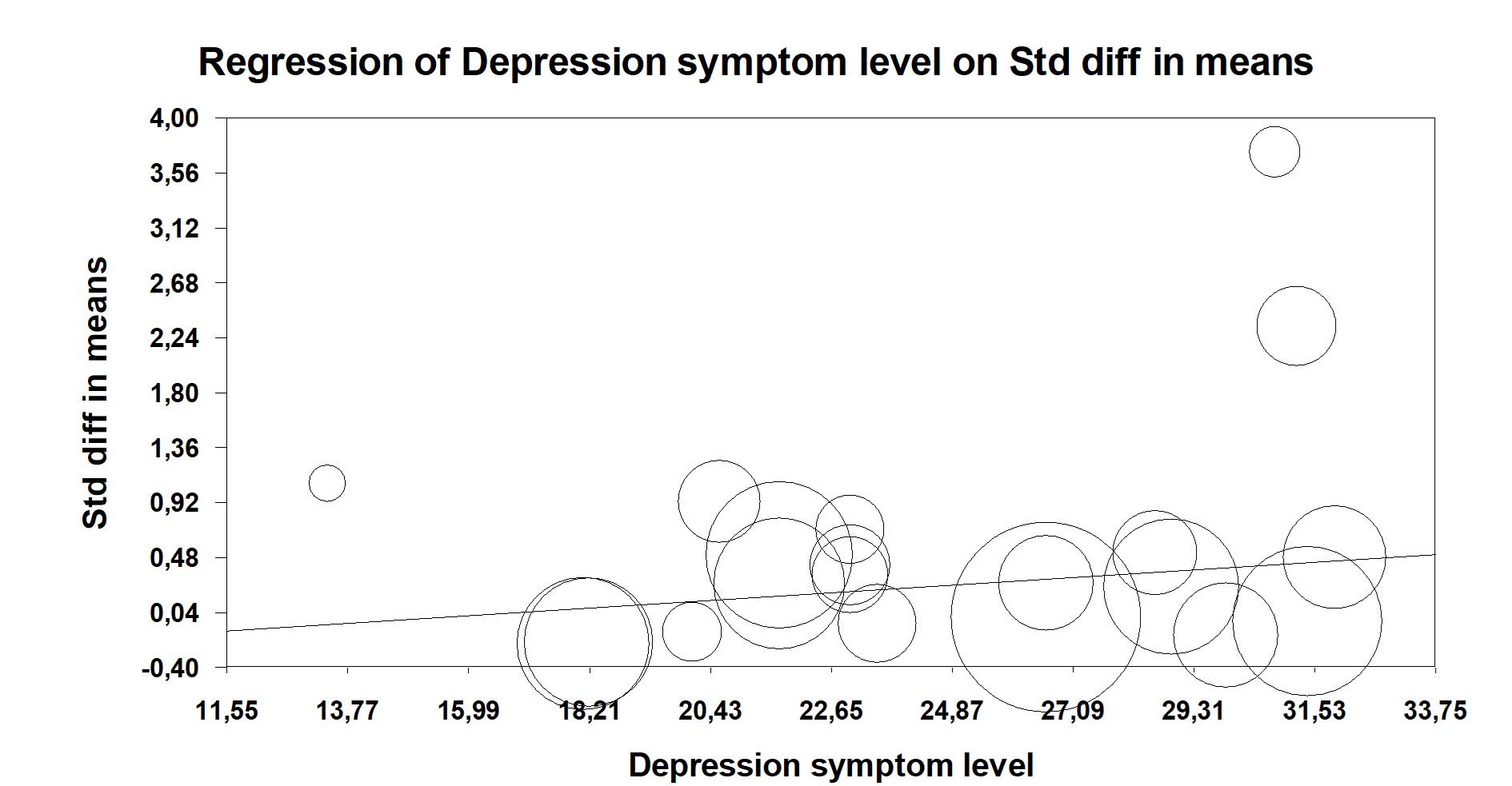
**Scatterplot showing results of meta-regression of baseline depression levels on glycemic control as outcome**. **High baseline depression score is associated with a greater reduction in HbA_1_c.**

**HbA1c**


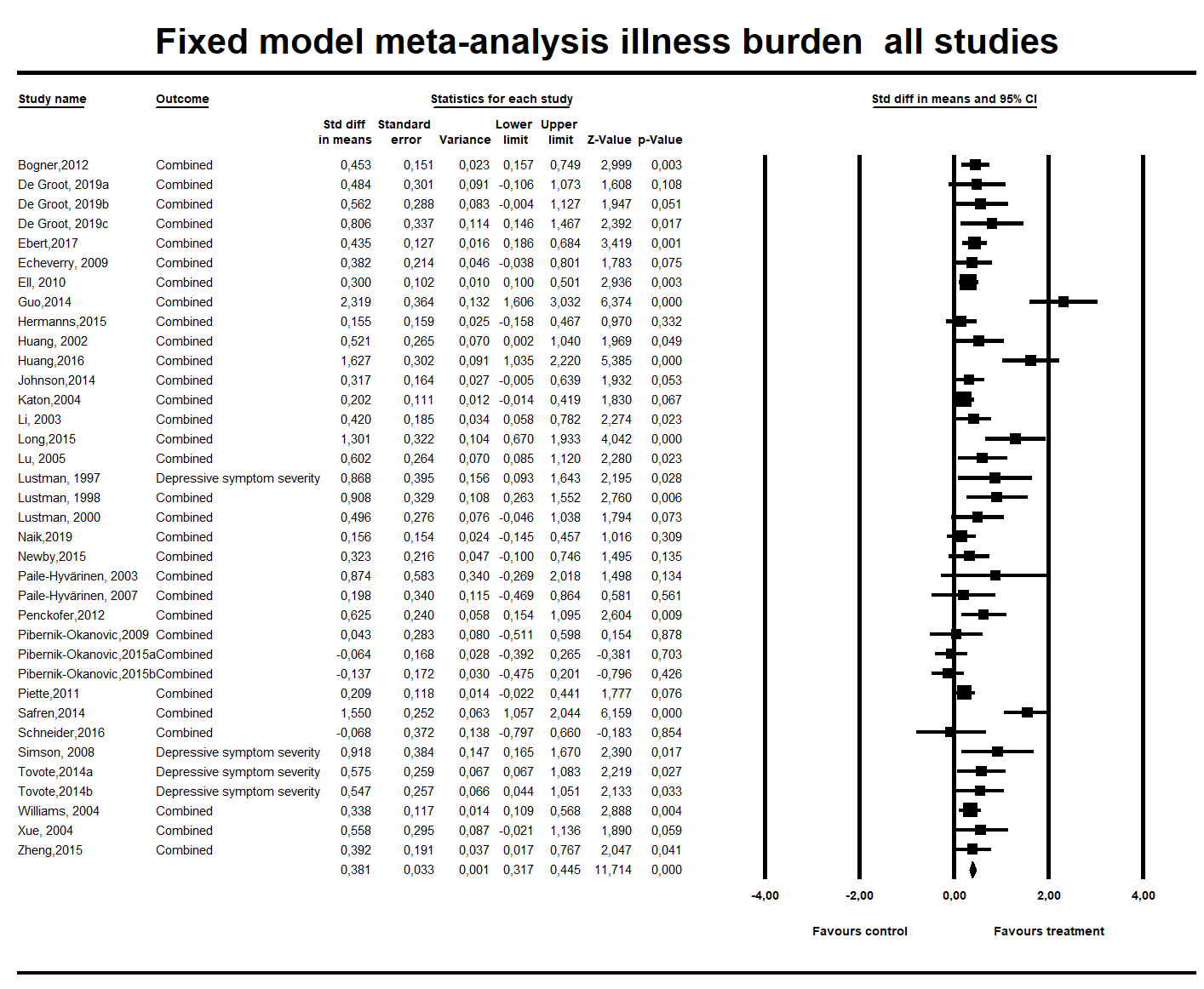


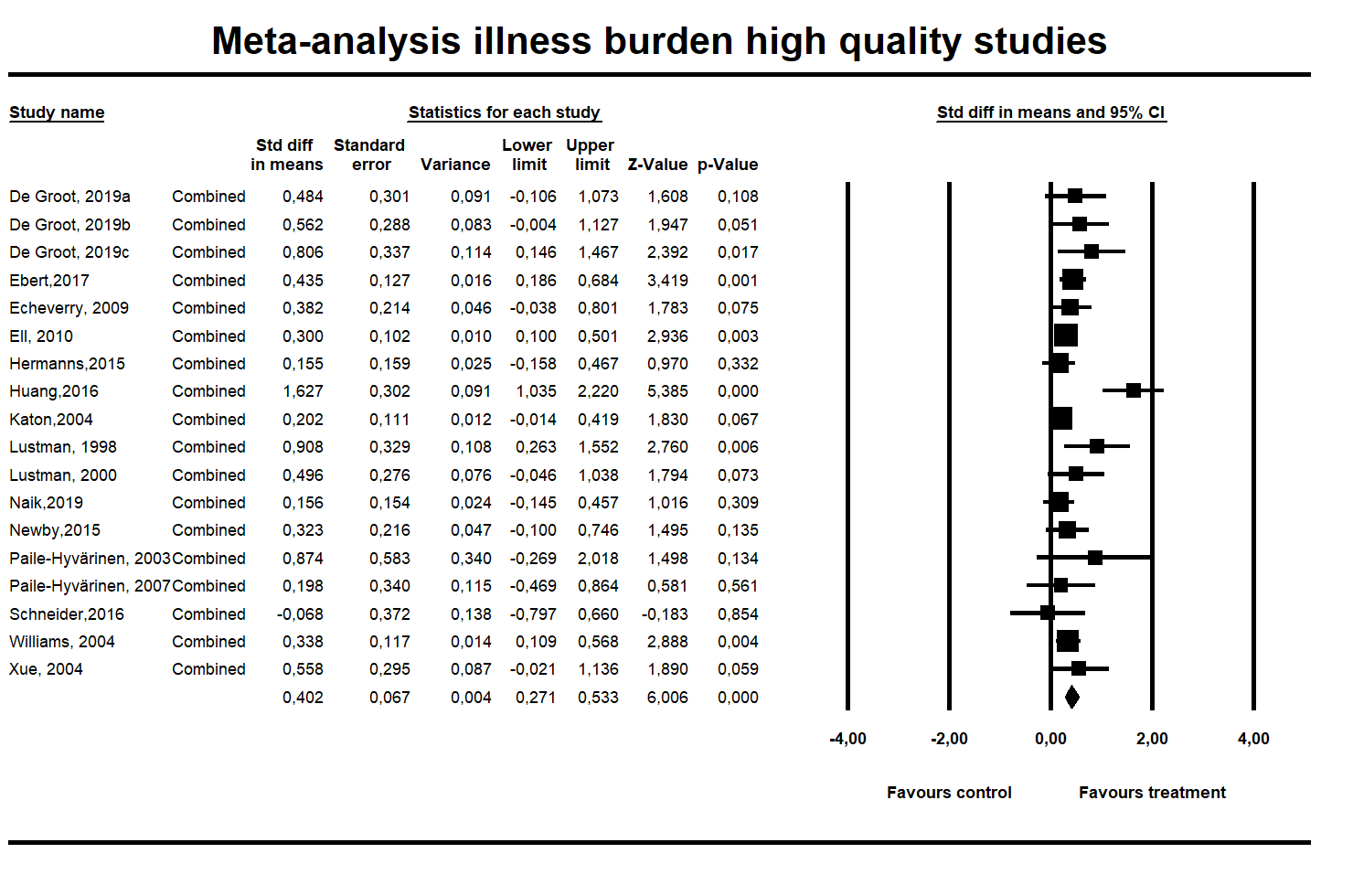


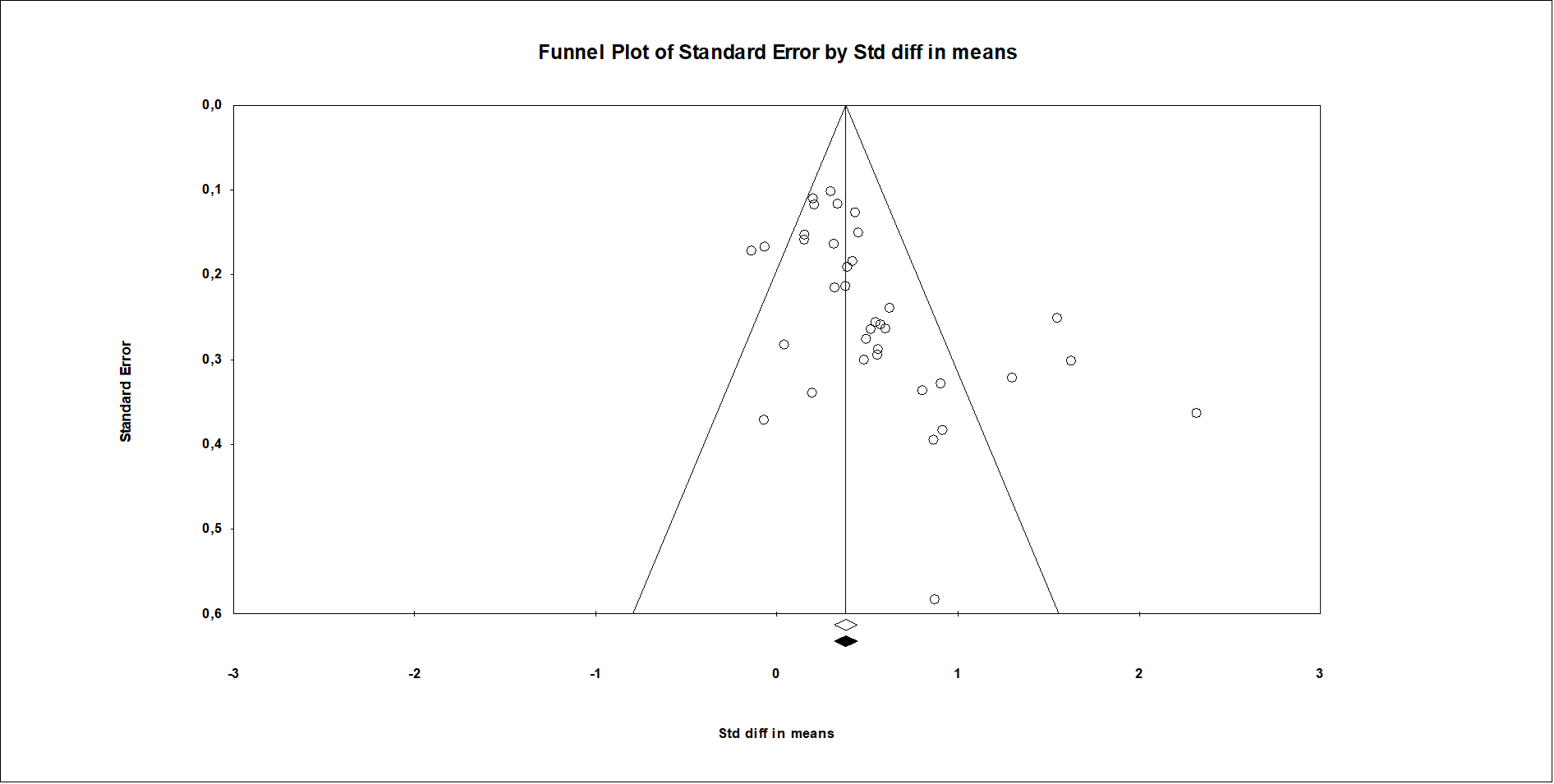


**Moderator analyses**

A moderator analysis was planned on the effect of add-on exercise on treatment outcome. However, the distribution of interventions in the group with and without add-on exercise was so dissimilar that the outcomes would not be valid and this moderator analysis was abandoned. A second intended moderator analysis aimed to explore the role of adherence focus in the treatment effect. As none of the subthreshold studies had adherence focus, they could not be included in the moderator analysis and there were only two studies with adherence focus in the MDD group, so this analysis was abandoned.

**References**

1 Van der Feltz-Cornelis CM, Nuyen J, Stoop C, Chan J, Jacobson AM, Katon W, Snoek F, Sartorius N: Effect of interventions for major depressive disorder and significant depressive symptoms in patients with diabetes mellitus: a systematic review and meta-analysis. General Hospital Psychiatry 2010;32:380-395.

2 Guy W: ECDEU assessment manual for psychopharmacology. US Department of Health, and Welfare 1976:534-537.

3 Busner J, Targum SD: The clinical global impressions scale: applying a research tool in clinical practice. Psychiatry (Edgmont) 2007;4:28.

4 Borenstein M, Hedges LV, Higgins JPT, Rothstein HR: ComprehensiveMeta-Analysis, Version 2. . Englewood (NJ), Biostat, 2005,

5 Hawley CJ, Gale TM, Smith PSJ, Jain S, Farag A, Kondan R, Avent C, Graham J: Equations for converting scores between depression scales (MÅDRS, SRS, PHQ‐9 and BDI‐II): good statistical, but weak idiographic, validity. Human Psychopharmacology: Clinical and Experimental 2013;28:544-551.

6 Choi SW, Schalet B, Cook KF, Cella D: Establishing a common metric for depressive symptoms: linking the BDI-II, CES-D, and PHQ-9 to PROMIS depression. Psychological Assessment 2014;26:513.

7 Leucht S, Fennema H, Engel RR, Kaspers-Janssen M, Szegedi A: Translating the HAM-D into the MADRS and vice versa with equipercentile linking. Journal of Affective Disorders 2018;226:326-331.

Knol MJ, Heerdink ER, Egberts AC, Geerlings MI, Gorter KJ, Numans ME, Grobbee DE, Klungel OH, Burger H: Depressive symptoms in subjects with diagnosed and undiagnosed type 2 diabetes. Psychosomatic Medicine 2007;69:300-305.

Anderson RJ, Freedland KE, Clouse RE, Lustman PJ: The prevalence of comorbid depression in adults with diabetes: a meta-analysis. Diabetes Care 2001;24:1069-1078.

Heinze G, Guizar-Sánchez D, Bernard-Fuentes N: Diabetes and Mental Health: From Distress to Depression; The Diabetes Textbook, Springer, 2019, pp 417-428.

Golden SH, Lazo M, Carnethon M, Bertoni AG, Schreiner PJ, Roux AVD, Lee HB, Lyketsos C: Examining a bidirectional association between depressive symptoms and diabetes. JAMA 2008;299:2751-2759.

Chireh B, Li M, D'Arcy C: Diabetes increases the risk of depression: a systematic review, meta-analysis and estimates of population attributable fractions based on prospective studies. Preventive Medicine Reports 2019:100822.

Salvi V, Grua I, Cerveri G, Menaccie C, F. B-A: The risk of new-onset diabetes in antidepressant users – A systematic review and meta-analysis. Plos One 2017

Bhattacharjee S, Bhattacharya R, Kelley GA, Sambamoorthi U: Antidepressant use and new-onset diabetes: a systematic review and meta-analysis. Diabetes Metabolism Research and Reviews 2013;29:273-284.

Spek V, Cuijpers P, Nyklíček I, Riper H, Keyzer J, Pop V: Internet-based cognitive behaviour therapy for symptoms of depression and anxiety: a meta-analysis. Psychological Medicine 2007;37:319-328.

Alanzi T, Istepanian RS, Philip N: An integrated model for cognitive behavioural therapy for mobile diabetes self-management system: 2014 36th Annual International Conference of the IEEE Engineering in Medicine and Biology Society, IEEE, 2014, pp 5393-5396.

van Bastelaar KM, Pouwer F, Cuijpers P, Twisk JW, Snoek FJ: Web-based cognitive behavioural therapy (W-CBT) for diabetes patients with co-morbid depression: design of a randomised controlled trial. BMC Psychiatry 2008;8:9.

Narita Z, Inagawa T, Stickley A, Sugawara N: Physical activity for diabetes-related depression: a systematic review and meta-analysis. Journal of Psychiatric Research 2019

Holt RI, De Groot M, Lucki I, Hunter CM, Sartorius N, Golden SH: NIDDK international conference report on diabetes and depression: current understanding and future directions. Diabetes Care 2014;37:2067-2077.
